# Metabolic dysfunction-associated steatotic liver disease-induced changes in the antioxidant system: a review

**DOI:** 10.1007/s00204-024-03889-x

**Published:** 2024-10-23

**Authors:** Gabriela Svobodová, Martin Horní, Eva Velecká, Iva Boušová

**Affiliations:** Department of Biochemical Sciences, Faculty of Pharmacy in Hradec Králové, Charles University, 500 05 Hradec Králové, Czech Republic

**Keywords:** Metabolic dysfunction-associated steatotic liver disease, Antioxidant enzyme, Glutathione, Expression, Catalytic activity

## Abstract

Metabolic dysfunction-associated steatotic liver disease (MASLD) is a heterogeneous condition characterized by liver steatosis, inflammation, consequent fibrosis, and cirrhosis. Chronic impairment of lipid metabolism is closely related to oxidative stress, leading to cellular lipotoxicity, mitochondrial dysfunction, and endoplasmic reticulum stress. The detrimental effect of oxidative stress is usually accompanied by changes in antioxidant defense mechanisms, with the alterations in antioxidant enzymes expression/activities during MASLD development and progression reported in many clinical and experimental studies. This review will provide a comprehensive overview of the present research on MASLD-induced changes in the catalytic activity and expression of the main antioxidant enzymes (superoxide dismutases, catalase, glutathione peroxidases, glutathione S-transferases, glutathione reductase, NAD(P)H:quinone oxidoreductase) and in the level of non-enzymatic antioxidant glutathione. Furthermore, an overview of the therapeutic effects of vitamin E on antioxidant enzymes during the progression of MASLD will be presented. Generally, at the beginning of MASLD development, the expression/activity of antioxidant enzymes usually increases to protect organisms against the increased production of reactive oxygen species. However, in advanced stage of MASLD, the expression/activity of several antioxidants generally decreases due to damage to hepatic and extrahepatic cells, which further exacerbates the damage. Although the results obtained in patients, in various experimental animal or cell models have been inconsistent, taken together the importance of antioxidant enzymes in MASLD development and progression has been clearly shown.

**Supplementary Information:**

The online version contains supplementary material available at 10.1007/s00204-024-03889-x.

## Introduction

Metabolic dysfunction-associated steatotic liver disease (MASLD), previously referred to as non-alcoholic fatty liver disease, is the most common liver disease, afflicting about 25–30% of the general population worldwide. Occurring in individuals who drink little to no alcohol, MASLD is characterized by hepatic fat accumulation (Korinkova et al. 2020; Perumpail et al. 2017; Rinella et al. 2023). This heterogenous and complex disease encompasses a broad clinical spectrum of liver abnormalities which vary in the severity of the injury and the resulting fibrosis. At one end of the spectrum, metabolic dysfunction-associated steatotic liver (hepatic steatosis, MASL) may progress to a more advanced state called metabolic dysfunction-associated steatohepatitis (MASH) characterized by significant hepatocyte damage, inflammation, and pericellular fibrosis, and further to cirrhosis (Friedman et al. 2018; Rinella et al. 2023). The pathogenesis of MASLD is complex, multifactorial, and not yet fully understood. Therefore, identification of the molecular mechanisms leading to MASLD-related fat accumulation, mitochondrial dysfunction, and oxidative stress is necessary to facilitate the development of specific interventions aimed at preventing the progression of hepatic steatosis. As no pharmacotherapy for MASLD has been approved at present, lifestyle changes represent the mainstay of therapy. Although lifestyle interventions have proven to be effective in MASLD management, achieving and sustaining such changes may be difficult for many patients (e.g. Finer 2022; Hannah and Harrison 2016)). In this review, we summarize the pathogenesis of MASLD with an emphasis on oxidative stress and MASLD-induced changes in antioxidant pathways.

## A brief introduction to the pathogenesis of MASLD

**Fig. 1:**
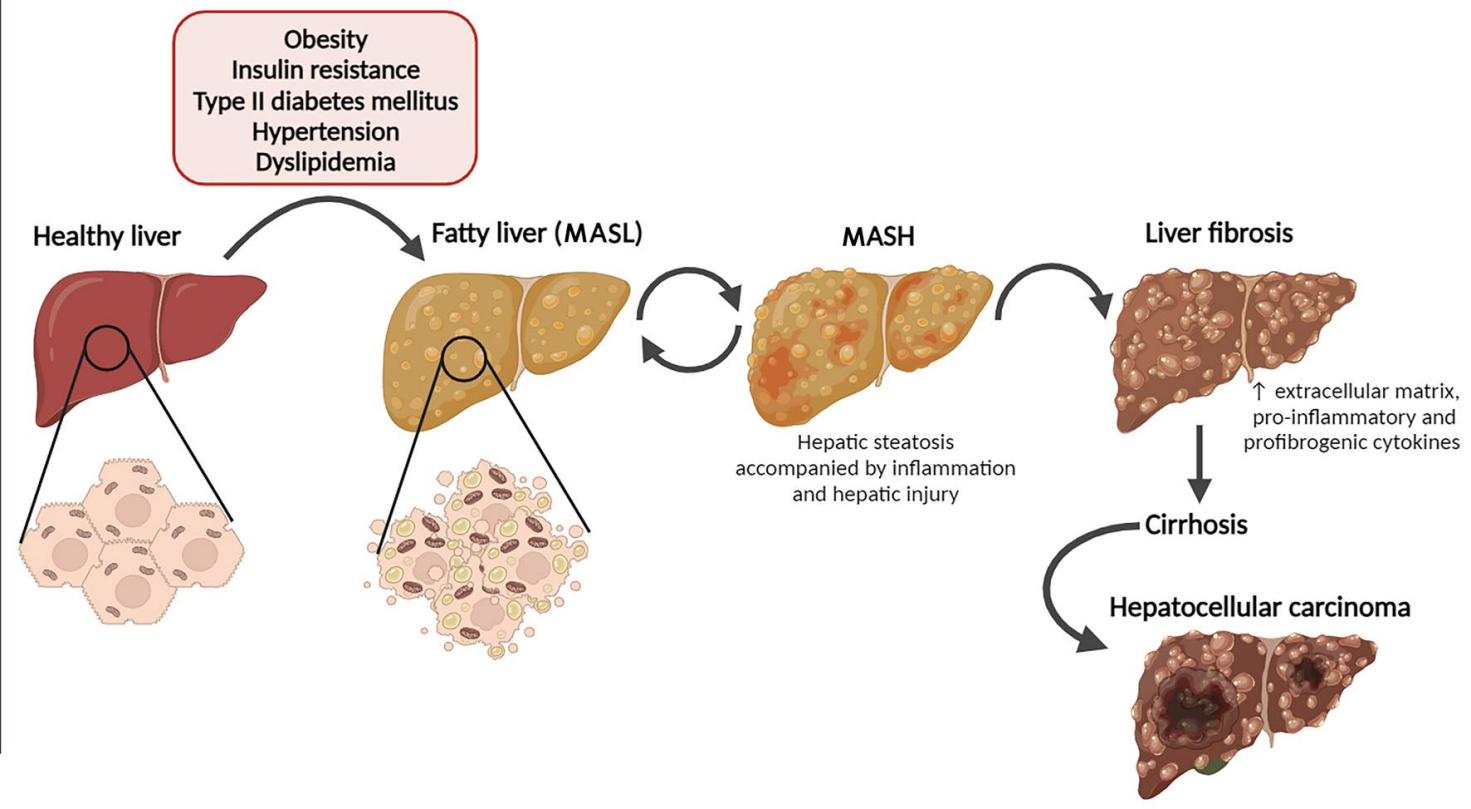
Progression of the metabolic dysfunction-associated steatotic liver disease. MASLD encompasses a broad clinical spectrum of liver damage. First, when exposed to some risk factors (e.g. insulin resistance, type 2 diabetes mellitus, obesity, hypertension, dyslipidemia) simple macrovesicular steatosis (MASL) develops in the liver of predisposed individuals. This form may progress to a more severe form of metabolic dysfunction-associated steatohepatitis (MASH), with combined inflammation, fibrosis, hepatocyte damage, and liver steatosis. Eventually, the disease may further advance to liver fibrosis, which carries the potential to escalate into life-threatening conditions such as liver cirrhosis and hepatocellular carcinoma

Based on the histological findings, MASLD is categorized as MASL and MASH. MASL is characterized by mixed macrovesicular and microvesicular hepatic steatosis (≥ 5% hepatocytes) which may be accompanied by mild inflammation, with the risk of progression to cirrhosis and liver failure considered minimal. In 10–20% of patients, hepatic steatosis progresses to a more advanced state called MASH, which is characterized by the presence of hepatic steatosis (≥ 5% hepatocytes), inflammation, and hepatocyte injury (ballooning) with or without fibrosis. MASH can progress to cirrhosis, liver failure, and even to hepatocellular carcinoma in rare cases (Fig. 1). Cirrhosis is the most frequent reason for a liver transplant (Hardy et al. 2016; Chalasani et al. 2018).

Pathogenesis of MASLD is closely related to metabolic syndrome. In patients with MASLD, features of metabolic syndrome (such as obesity, type 2 diabetes, insulin resistance, hypertension, and dyslipidemia) are highly prevalent and increase the risk of MASLD development (Chalasani et al. 2018). According to the currently accepted multiple-hit theory, several simultaneously acting “hits” (e.g. insulin resistance, excessive secretion of adipokines, unhealthy lifestyle, gut dysmicrobiosis) act together on a genetically predisposed person to induce MASLD (Buzzetti et al. 2016). According to this theory, oxidative stress plays an important role in MASLD progression as the starting point of hepatic damage (Masarone et al. 2018).

In following subsection, the effect of hepatic oxidative stress on the pathogenesis of MASLD will be discussed. Genetic and epigenetic factors determining MASLD have been recently reviewed by Jonas and Schurmann (Jonas and Schurmann 2021), and the role of gut microbiota in mediating MASLD development was summarized by Campo et al. (Campo et al. 2019).

### Oxidative/nitrosative stress and mitochondrial dysfunction

**Fig. 2:**
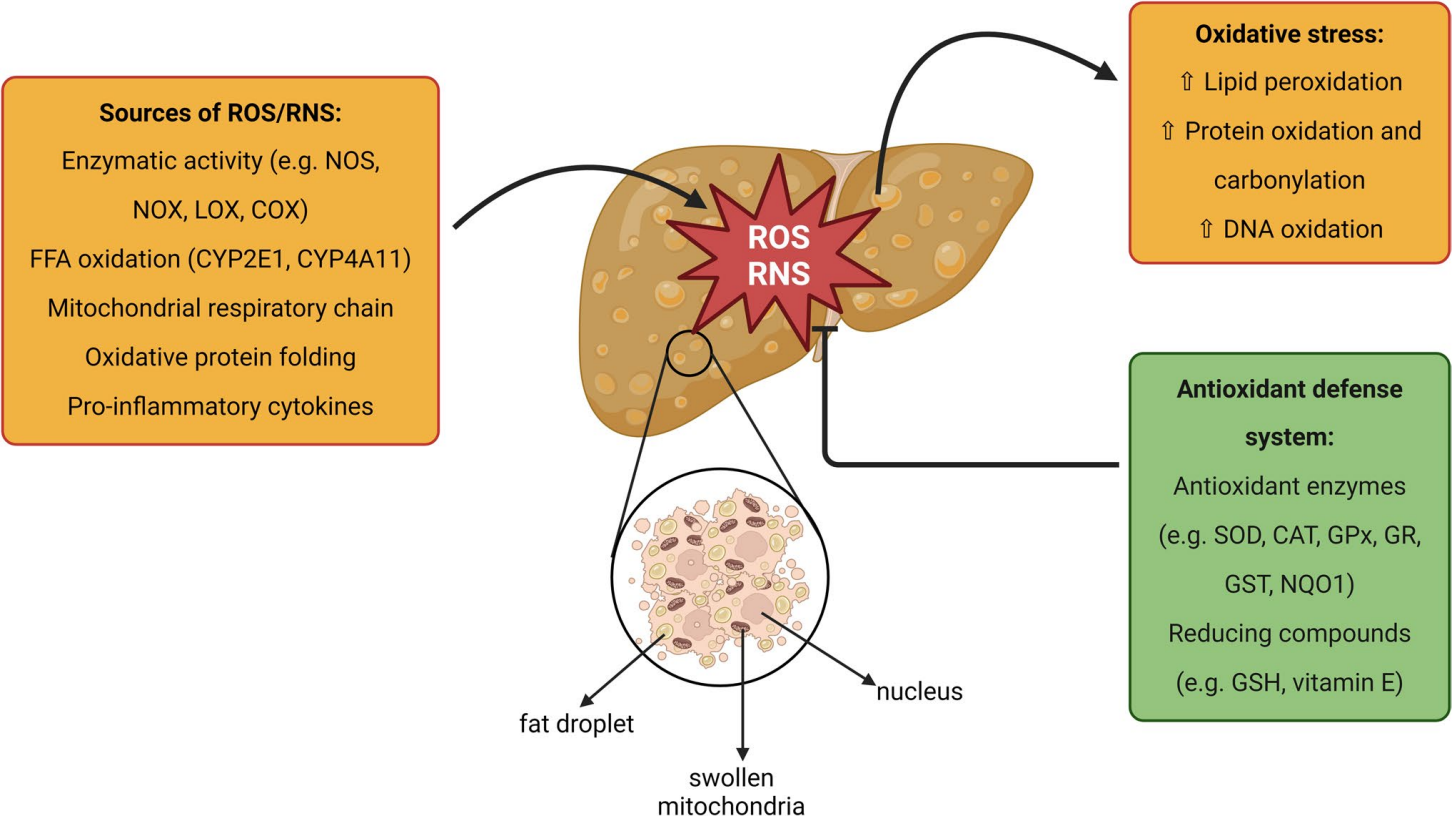
Oxidative and nitrosative stress in MASLD. Oxidative and nitrosative stress are recognized as the significant contributors to hepatocellular injury in MASLD and the critical drivers of the transition from MASL to MASH. In the cell, reactive oxygen species (ROS) and reactive nitrogen species (RNS) are generated by several mechanisms comprising catalytic activity of various enzymes (e.g. cyclooxygenase, lipoxygenase, nitric oxide synthase, NADPH oxidase), oxidative protein folding in endoplasmic reticulum, ω-oxidation of free fatty acids catalyzed by cytochrome P450 (CYP) 2E1 and CYP4A11 in peroxisomes and microsomes, the effect of pro-inflammatory cytokines, and mitochondrial respiratory chain. Oxidative and nitrosative stress are associated with widespread protein oxidation and carbonylation, lipid peroxidation, and DNA oxidation, which reduces the antioxidative capacity and shifts the intracellular redox status toward an oxidized state. Expression and/or activity of antioxidant enzymes as well as intracellular levels of glutathione can be altered. Abbreviations: *CAT* catalase; *COX* cyclooxygenase; *GPx* glutathione peroxidase; *GR* glutathione reductase; *GSH* glutathione; *GST* glutathione S-transferase; *LOX* lipoxygenase; *NOS* nitric oxide synthase; *NOX* NADPH oxidase; *NQO1* NAD(P)H quinone oxidoreductase; *SOD* superoxide dismutase

Oxidative/nitrosative stress is caused by an imbalance between the generation of reactive oxygen/nitrogen species (ROS/RNS) and antioxidant defenses, which leads to cellular and tissue damage. It may occur through both the increasing ROS/RNS production and the dysfunction of the antioxidant system (Delli Bovi et al. 2021). Oxidative/nitrosative stress is recognized as one of the significant contributors to hepatocellular injury in MASLD and a critical driver of the transition from MASL to MASH (Prasun et al. 2021). In the cell, ROS/RNS are generated by several mechanisms comprising the catalytic activity of various enzymes (e.g. amino acids oxidases, cyclooxygenase, lipoxygenase, nitric oxide synthase, xanthine oxidase, NADPH oxidase), oxidative protein folding in the endoplasmic reticulum (ER), the oxidation of free fatty acids (FFAs) in peroxisomes and microsomes, through the effect of pro-inflammatory cytokines, and in the mitochondrial respiratory chain. Oxidative/nitrosative stress is associated with widespread protein and lipid (per)oxidation, which reduces antioxidative capacity and shifts the intracellular redox status toward an oxidized state (Reiniers et al. 2014) (Fig. 2). 3-Nitrotyrosine (a marker of protein nitration) and malondialdehyde (a marker of lipid peroxidation), were shown to be significantly increased in patients with MASLD/MASH, a condition which correlated with the observed severity of liver oxidative injury (Musso et al. 2007; Sanyal et al. 2001), as well as in high-fat diet (HFD)-fed rats (Reiniers et al. 2014).

Mitochondrial dysfunction is characterized by various degrees of ultrastructural mitochondrial damage, abnormal morphologic changes, increased permeability of the outer and inner membranes, impaired mitochondrial β-oxidation, respiratory chain activity reduction, ROS overproduction, ATP depletion, and oxidative stress-mediated deletions of mtDNA inducing apoptosis (Ramanathan et al. 2022). Mitochondrial dysfunction of hepatic cells was described in MASLD (Jha and Mitra Mazumder 2019; Sanyal et al. 2001) and contributes significantly to oxidative stress in the liver. Directly and indirectly, ROS contribute to stellate/Kuppfer cell activation and chronic inflammatory response with up-regulation of pro-inflammatory cytokines (e.g. tumor-necrosis factor α (TNF-α), interleukin-1α and 6 (IL-1α and IL-6)) via activation of nuclear factor NF-κB (Wang et al. 2009). In FFA-treated HepG2 cells, the NF-κB-dependent expression of TNF-α was at least partially mediated by cathepsin B-dependent lysosomal membrane destabilization. The lysosomal destabilization and cathepsin B release to the cytosol has been confirmed in a murine in vivo model as well as in human MASLD (Feldstein et al. 2004).

Hepatic triglyceride accumulation resulting from a complex interplay between adipose tissue and liver is a hallmark of MASLD, with insulin resistance a major pathogenic mechanism in this context. When adipose tissue becomes resistant to the anti-lipolytic effect of insulin, degradation of triglycerides proceeds and leads to a massive release of FFAs into systemic circulation (Svegliati-Baroni et al. 2019). Indeed, plasmatic levels of FFAs were shown to be significantly elevated in MASLD patients compared to healthy controls (Feng et al. 2017). The FFAs are then taken up by the liver, where they accumulate in the form of triglycerides. Moreover, increased levels of ketone bodies (e.g. acetoacetate, β-hydroxybutyrate, acetone) were found in the plasma of MASLD patients. Synthesis of ketone bodies may be initially increased in MASLD as a mechanism to safely manage excess triglyceride‐derived acetyl‐CoA via nonoxidative disposal to limit oxidative stress, a mechanism which becomes disturbed as MASLD progresses (Post et al. 2021).

In patients with MASLD and obesity, the increased activity and expression of microsomal cytochrome P450 (CYP) 2E1 and CYP4A11 was observed, with this increase showing a significant correlation with elevated levels of circulating ketone bodies and FFAs, high-density lipoprotein, triglycerides, insulin, and insulin resistance (Aljomah et al. 2015; Gao et al. 2020). These enzymes are involved in the metabolism of medium- and long-chain fatty acids (e.g. arachidonate and palmitate) and catalyze their ω-hydroxylation. Under normal conditions, FFAs either enter the mitochondria and undergo β-oxidation or are esterified to triglycerides and subsequently transported from hepatocytes in very low-density lipoproteins. During the MASLD-induced mitochondrial dysfunction, the microsomal CYP2E1- and CYP4-mediated ω-hydroxylation of FFAs is markedly increased (Daly 2013; Hardwick 2008). This process is accompanied by excessive ROS generation; CYP2E1 is a well-known “leaky” enzyme (due to the constitutive high spin form of this enzyme) with a unique ability to produce ROS and other radical intermediates during the catalytic cycle (Harjumaki et al. 2021). In the case of CYP4A11, the exposure of HepG2 cells to a mixture of palmitic and oleic acid resulted in elevation of mRNA as well as protein expression of CYP4A11 and an increase in intracellular ROS content (Gao et al. 2020). In addition, oxidative stress leads to the activation of the NF-κB signaling pathway and the synthesis of proinflammatory cytokines, thus ROS production by CYP4A11 metabolism of FFA relates to inflammatory reactions (Gao et al. 2020). Moreover, chronic oxidative stress generated through CYP2E1 induction was shown to interfere with the insulin signaling cascade via a decrease in tyrosine phosphorylation of insulin receptor substrates 1 and 2. Chronic oxidative stress thus contributes to insulin resistance development (Schattenberg et al. 2005). One of the key regulators of fatty acid oxidation is transcription factor peroxisome proliferator-activated receptor-α (PPARα). Interaction between the PPARα pathway and the enzymes CYP4A and CYP2E1 has been described (Ip et al. 2003). CYP4A genes are under the partial control of PPARα, and their induction was shown to be dependent on PPARα expression in diabetic rats (Kroetz et al. 1998). Moreover, Cyp2e1-null mice fed with ethanol developed centrilobular fat accumulation with upregulation of PPARα, suggesting an interplay between CYP2E1 and PPARα-mediated fatty acid homeostasis (Wan et al. 2001). However, the clinical relevance of these observations has yet to be proved.

Given the pivotal role of oxidative stress in the pathogenesis of MASLD, it is essential to investigate the consequent alterations in the antioxidant defense system, particularly during the heightened production of ROS/RNS throughout the progression of the disease.

## MASLD-induced changes in antioxidant system

To survive in an oxygen-rich environment, aerobic organisms had to develop efficient defense systems of enzymatic and non-enzymatic antioxidants to control the cellular levels of ROS to maintain redox homeostasis (Miao and St Clair 2009; Tsang et al. 2014; Zelko et al. 2002). Among the main antioxidant enzymes involved in ROS removal belong superoxide dismutase (SOD), catalase (CAT), glutathione peroxidase (GPx), and peroxiredoxins (Halliwell 2024); moreover, drug-metabolizing enzymes NAD(P)H:quinone oxidoreductase 1 (NQO1) and glutathione S-transferase (GST) can participate in antioxidant defense as well (Boušová and Skálová 2012; Ross and Siegel 2021). The function of the antioxidant system is supported by recycling and synthesizing substrates of antioxidant enzymes by auxiliary enzymes, such as glutathione reductase (GR) and glucose 6-phosphate dehydrogenase (Couto et al. 2016). The participation of transition metal ions in the free radical generation is protected by several metal-sequestering proteins (e.g. transferrin, ferritin, ceruloplasmin) and some low-molecular-weight antioxidants (e.g. glutathione, ascorbic acid, α-tocopherol) (Jomova et al. 2024; Szarka et al. 2012). The low-molecular-weight antioxidants also possess the ability to scavenge ROS; during this process are oxidized and sometimes can be recycled back to their reduced forms, e.g. oxidized glutathione (GSSG) by GR, α-tocopheryl radical by ubiquinol, ascorbate or NQO1, ascorbyl radical by ascorbic acid reductase (Fujii et al. 2022; Halliwell 2024).

ROS are generated as byproducts of substrates oxidation or during aerobic respiration in mitochondria (García-Ruiz and Fernández-Checa 2018; Tsang et al. 2014). An imbalance between high levels of ROS and low cellular antioxidant defenses is referred to as “oxidative stress” (Halliwell 2014). Although certain ROS concentrations are necessary for the normal physiological functions of living aerobic organisms, as they are involved in cell signaling pathways and disposal of pathogens (Miao and St Clair 2009; Zelko et al. 2002), elevated concentrations of ROS cause oxidation as well as the loss of normal functions of many endogenous macromolecules such as lipids, nucleic acids and proteins, resulting in the development of various diseases (Mondola et al. 2016; Tsang et al. 2014; Zelko et al. 2002). Decreased antioxidant defense is a major factor which promotes oxidative stress in patients with MASLD (Shin et al. 2018). Moreover, the impaired activity and/or expression of antioxidant enzymes in MASLD carry several significant clinical implications. The diminished function of key antioxidant enzymes and the overproduction of ROS/RNS can disrupt insulin signaling pathways, exacerbating hepatic lipid accumulation. This results in increased lipid peroxidation, causing dysfunction in mitochondria and the ER. Such organelle damage can trigger apoptosis and hepatocyte cell death. Furthermore, reduced antioxidant enzyme activity promotes pro-inflammatory signaling pathways and activates hepatic stellate cells, driving liver fibrosis progression. Chronic oxidative stress also causes DNA damage, contributing to genomic instability and mutations, which elevate the risk of malignant transformation and the development of hepatocellular carcinoma in MASLD patients (Arroyave-Ospina et al. 2021; Ma et al. 2021). Also, oxidative damage in MASLD is closely linked to cardiovascular complications, including atherosclerosis, hypertension, and heart failure, which are the leading cause of mortality in MASLD patients (Gutiérrez-Cuevas et al. 2021; Ramírez-Mejía et al. 2024). Additionally, emerging evidence suggests that chronic systemic oxidative stress, inflammation, and insulin resistance contribute to neuroinflammation and neuronal damage, increasing the risk of Alzheimer’s disease (Patel and Edison 2024).

Numerous data regarding the relationship between MASLD and changes in the antioxidant system have been reported, although many results are inconsistent mainly due to variability in models used (patients, experimental animals, cells). The present review summarizes and categorizes the available data to highlight the importance of antioxidant systems for the MASLD development and progression. Therefore, the following subsections will provide an in-depth analysis of alterations in the main antioxidant enzymes, including SODs, CAT, GPxs, GSTs, GR, and NQO1, as well as in the non-enzymatic antioxidant GSH observed in MASLD patients as well as in various MASLD/MASH experimental models. Additionally, the final subsection will summarize the potential therapeutic effects of vitamin E in restoring the function of these antioxidant enzymes and glutathione levels altered by this disease.

### MASLD and superoxide dismutase family

Superoxide dismutases (SODs) represent the first and the most important line of antioxidant enzyme defense against ROS. SODs efficiently catalyze the dismutation of highly reactive superoxide, which is primarily generated within the mitochondrial matrix as a product of single electron reduction of molecular oxygen through the electron transport chain (Miao and St Clair 2009; Tsang et al. 2014; Zelko et al. 2002). Three eukaryotic SODs have been characterized based on their biochemical and molecular properties: the cytosolic copper-zinc (Cu–Zn) dimeric form (known as SOD1), the mitochondrial tetrameric manganese (Mn) form (known as SOD2), and the extracellular tetrameric Cu–Zn form (known as SOD3). All of these isoenzymes are involved in the conversion of superoxide to molecular oxygen and hydrogen peroxide (Miao and St Clair 2009; Mondola et al. 2016; Tsang et al. 2014; Zelko et al. 2002).

The expression profiles of SOD1 and SOD2 increase toward adulthood in the lung and liver (Asikainen et al. 1998), but, surprisingly, considerably higher plasma levels of SOD3 have been detected in children as compared to adults (Adachi et al. 2000). Carriage of the SOD2 C47T (rs4880) polymorphism has been associated with steatosis as well as more advanced fibrosis in MASH (Al-Serri et al. 2012). This polymorphism is responsible for a rise in SOD enzymatic activity used for detoxifying superoxide anion radicals, thus the resultant phenotypes produce a greater number of toxic intermediates as well as hydrogen peroxide. This condition has been found to increase a patient’s susceptibility to many diseases, including MASH (Huang et al. 2014, 2021). A study of the frequency of 1183 T/C polymorphism of SOD2 in Japanese MASH patients revealed a higher incidence of the T/T genotype in the mitochondrial targeting sequence of this gene, resulting in the less effective targeting of Mn-SOD to mitochondria in these patients (Namikawa et al. 2004).

Many inconsistent results regarding changes in the expression and/or activity of SODs in association with MASLD/MASH have been published (Table S1). The serum activity of SOD was significantly elevated in patients suffering from early MASLD and advanced MASLD as compared to controls, indicating an adaptive reaction to the increased production of ROS during MASLD (Świderska et al. 2019). Higher plasmatic SOD activity was also observed in MASLD subjects with high intrahepatic fat content (Monserrat-Mesquida et al. 2020). Indeed, the specific activity of Cu/Zn-SOD was elevated by 60% in liver biopsies obtained from MASLD patients than in control livers; contrarily, no significant differences in hepatic Mn-SOD nor in erythrocytic Cu/Zn-SOD activity were observed (Perlemuter et al. 2005). However, in a study focused on redox imbalance in MASLD patients, erythrocytic SOD activity was significantly higher in patients with MASLD as compared to healthy controls (Asghari et al. 2020). Elevated SOD activity was also observed in HFD-fed mice (Boland et al. 2018; Marinho et al. 2018) and rats (Barbosa et al. 2021; Song et al. 2017; Tsai et al. 2016) as compared to control groups, respectively. Similarly, a high-fat diet, a high-fructose diet, and an iron-rich diet all caused a significant elevation in SOD activity in experimental animals as compared to respective controls (Jarukamjorn et al. 2016; Valenzuela et al. 2018).

Surprisingly, some studies reveal a marked decrease in serum or hepatic SOD activity in patients suffering from MASH as compared to healthy controls (Koruk et al. 2004; Videla et al. 2004). In a study by Kanoni et al., MASLD-obese patients also displayed a significant reduction in erythrocytic SOD activity as compared to a control group (Kanoni et al. 2021). In several studies with HFD-fed experimental animals, hepatic and plasma/serum SOD activity significantly declined in comparison to standard chow diet-fed controls (Hou et al. 2016; Lou et al. 2024; Murakami et al. 2018; Nayan et al. 2021). Similarly, Wang et al. reported substantially decreased SOD2 activity in a rat MASH model as compared to a control group (Wang et al. 2017). Interestingly, total SOD activity as well as SOD2 activity, were reported to be decreased, whereas SOD1 activity was shown to have increased in a mouse MASLD model as compared to controls (Wu et al. 2015). Markedly reduced hepatic SOD activity has also been observed in many other diet-induced animal MASLD/MASH models, such as animals with a methionine-choline deficient diet, an iron-rich diet, monosodium glutamate (MSG)-induced obesity, lipopolysaccharide-induced liver injury as well as CCl_4_-induced liver injury (Alfarisi et al. 2020; Carvalho et al. 2018; Dai et al. 2014; Dong et al. 2018; Jung and Kim 2013; Li and Lu 2018; Lu et al. 2019; Nosrati et al. 2010; Santos-López et al. 2016; Yang et al. 2016). Indeed, reduced SOD activity was reported in HepG2 cells treated with fatty acids as compared to controls (Li et al. 2021, 2020; Su et al. 2018; Xia et al. 2018).

In several studies, the activity of SOD was not influenced by the onset of MASLD/MASH. In children with MASLD/MASH, results have shown no significant differences in blood SOD activity as compared to healthy age-matched controls (Nobili et al. 2005). Indeed, SOD activity was comparable in the red blood cells of MASLD patients and healthy subjects, although activity was significantly higher as compared with a patient suffering from chronic viral hepatitis (Kumar et al. 2013). Moreover, no significant changes were reported in SOD activity in mice on HFD or in other animal models of MASLD as compared to healthy controls (Matouskova et al. 2015; Mendes et al. 2018; Nunes-Souza et al. 2016; Su et al. 2018; Vornoli et al. 2014; Xu et al. 2017). Neither sex-related nor diet-related changes in liver SOD activity were shown in cafeteria diet-fed male and female mice as compared to respective standard chow diet-fed controls (Gasparin et al. 2018).

Regarding protein expression of SOD, MASH patients displayed elevated SOD2 protein levels as compared to control subjects (Shearn et al. 2017). Conversely, reduced protein expression of SOD has been reported in several animal species as well as in cellular models of MASLD. Significantly decreased protein expression of SOD1 and SOD2 was reported in HFD-fed mice (Chen et al. 2015) and rats (Li et al. 2021) as compared to controls, respectively. Likewise, recent studies have shown that the protein level of SOD2 is significantly decreased in the hepatic tissue of mice fed a Western diet (Jakubek et al. 2024) and the reduced protein expression of all SOD isoforms in the steatotic liver of diabetic mice can be restored by the administration of extracellular SOD, which also leads to a significant reduction in ROS levels (Nam et al. 2023). Significantly decreased protein expression of SOD1 and SOD2 was reported in HepG2 cells treated with palmitic acid (Chen et al. 2015); similarly, diminished protein levels of SOD2 were reported in HepG2 cells treated with oleic acid (Sharma et al. 2020).

Regarding gene expression, SOD3 mRNA expression was found to be upregulated in patients with advanced MASLD as compared to those with mild MASLD; moreover, differently methylated regions (both hypomethylated and hypermethylated) were found in the Sod3 gene (Hotta et al. 2018). Patients with MASLD showed induced mRNA expression of SOD1 as compared to controls (Kohjima et al. 2007). Elevated mRNA expression of SOD1 and SOD2 was observed also in mice fed a high-fat, high-fructose diet in comparison to control subjects (Jarukamjorn et al. 2016).

In contrast, measurement of hepatic SOD1 gene expression showed that the mRNA level of liver SOD1 was significantly decreased in subjects with cirrhosis secondary to MASH as compared to subjects with cirrhosis secondary to primary biliary cirrhosis as well as with healthy controls, respectively (Sreekumar et al. 2003). Significantly decreased mRNA expression of SOD1 was observed in the white adipose tissue of KK-A^y^ mice compared to C57BL/6 control mice (Furukawa et al. 2004). In high-saturated/high-cholesterol diet-fed rats, depletion of SOD1 as well as depletion of SOD2 mRNA expression was observed in the liver as compared to controls (Santos-López et al. 2016). Similarly, a significant reduction in the mRNA level of SOD2 (Nayan et al. 2021; Park et al. 2016; Xia et al. 2016, 2018) as well as in relative gene expression of SOD1 and SOD2 (Krishnan et al. 2017) was reported in various animal models of MASLD compared to controls, respectively. Recent research also reported significantly decreased mRNA expression of SOD in the liver of mice fed a HFD and carbohydrate-rich water (Rosas-Campos et al. 2024). Decreased mRNA expression of SOD1 or SOD2 was also reported in HepG2 cells treated with fatty acids (Jain et al. 2018; Xia et al. 2018).

Finally, no changes were observed in relative mRNA levels of SOD1 and SOD2 in conventional MASH patients (Nagaya et al. 2015) as well as in cafeteria diet-fed male and female mice (Gasparin et al. 2018) as compared to controls, respectively. No significant changes were observed in SOD2 mRNA levels in rats (Tanaka et al. 2014; Zhu et al. 2017), nor were any changes detected in SOD1 mRNA levels in HFD-fed mice (Du et al. 2016) as compared to controls, respectively.

### MASLD and catalase

Catalase (CAT), the oldest known and first-discovered antioxidant enzyme, plays an important role in defending cells against oxidative damage by decomposing two molecules of hydrogen peroxide into two molecules of water and one molecule of molecular oxygen (2H_2_O_2_ → 2H_2_O + O_2_) (Alfonso-Prieto et al. 2009; Glorieux and Calderon 2017; Shin et al. 2018). CATs have been classified into three groups based on their structure and function: monofunctional heme-containing catalases, heme-containing catalase-peroxidases, and manganese-containing catalases (Glorieux and Calderon 2017). The highest levels of CAT have been found in the liver, kidney, and blood, mainly located in peroxisomes in an active tetrameric conformation (Glorieux and Calderon 2017; Ho et al. 2004; Shin et al. 2018), although the existence of a tetrameric cytosolic CAT has been reported (Middelkoop et al. 1993).

CAT has a lower affinity for H_2_O_2_ as compared to glutathione-peroxidase (GPx), another enzyme responsible for the degradation of H_2_O_2_. However, as the concentration of H_2_O_2_ increases, CAT degrades H_2_O_2_ with higher efficiency, resulting in the prevention of more severe forms of MASLD. If H_2_O_2_ is not removed by CAT, the extremely reactive hydroxyl radical is generated in the Fenton reaction, resulting in additional oxidative stress (Shin et al. 2018). Regarding gene polymorphisms, it was reported that the most important single nucleotide CAT polymorphism −262C > T (rs1001179) may reduce the enzymatic activity of CAT, and the CAT mutant T allele remains the highest independent risk factor for MASH development (Huang et al. 2021; Kosmalski et al. 2023).

Clinical studies examining the mRNA/protein expression and/or specific activity of CAT enzymes which occur in MASL have shown conflicting results (Table S2). One study conducted in human subjects showed that CAT expression differs according to the stage of MASLD, with expression generally increased in the early stage of MASLD and decreased in the terminal stage of MASH (Shin et al. 2018).

Several studies have demonstrated significantly elevated CAT activity in the liver (Perlemuter et al. 2005) and in the plasma (Monserrat-Mesquida et al. 2020) of MASLD patients. Similarly, increased CAT activity was reported in the livers of adult as well as pediatric MASH patients as compared to age-matched controls (Baker et al. 2010; Moya et al. 2015). In high-fructose diet-fed rats, CAT activity was significantly increased in the liver as compared to a control group (Carvalho et al. 2018). Markedly increased hepatic CAT activity has also been observed in other diet-induced MASLD/MASH animal models, such as animals with a high-fructose diet, a high-fat/high fructose diet, and an iron-rich diet (Barbosa et al. 2021; Jarukamjorn et al. 2016; Valenzuela et al. 2018). Elevation of CAT activity was also reported in rat hepatoma steatotic cells as compared to their respective controls (Vecchione et al. 2016).

Interestingly, some studies have revealed significantly reduced CAT activity in MASH patients as compared to patients with simple fatty liver and healthy subjects, respectively (Videla et al. 2004), as well as in MASLD patients in comparison to the healthy controls (Das et al. 2008; Kosmalski et al. 2023; Kumar et al. 2013; Yesilova et al. 2005). Moreover, Das et al. found that excessive production of superoxide radicals can inactivate catalase (Das et al. 2008). Świderska et al. reported that serum CAT activities in early and advanced MASLD groups were decreased as compared to healthy controls (Świderska et al. 2019), whereas a research study by Perlemuter et al. showed no differences in CAT activity in erythrocytes (Perlemuter et al. 2005). Likewise, results obtained in various diet-induced MASLD/MASH rodent models revealed a marked reduction in CAT activity in the serum as well as the liver of these animals as compared to respective controls (Dai et al. 2014; Gasparin et al. 2018; Jorgačević et al. 2014; Korish and Arafah 2013; Murakami et al. 2018; Santos-López et al. 2016; Thomàs-Moyà et al. 2008; Yoshioka et al. 2010; Zakaria et al. 2021). In addition, HepG2 cells treated with the combination of fatty acids displayed a decline in CAT activity (Su et al. 2018; Xia et al. 2018).

On the other hand, several studies have reported no significant influence of MASLD/MASH on CAT activity. In MASLD and MASH patients, unchanged CAT activity was observed in both groups as compared to controls (Li et al. 2018; Perlemuter et al. 2005). A study conducted by Boland et al. showed that ob/ob mice fed both high-*trans*-fat, high-fructose and high-cholesterol (AMLN) diet as well as a standard chow diet had the same level of CAT activity, while AMLN diet-fed mice had markedly increased oxidative DNA damage (Boland et al. 2018). In diet-induced MASLD rodent models, hepatic CAT enzymatic activity showed a non-significant difference as compared to respective standard chow diet-fed animals (Jung and Kim 2013; Mendes et al. 2018; Noeman et al. 2011; Nunes-Souza et al. 2016; Ortenzi et al. 2024; Vornoli et al. 2014; Wu et al. 2015). In MSG-obese mice, the specific activity of CAT remained unchanged in the liver, while it was reduced in the small intestine (Matouskova et al. 2015).

A study by Li et al. examined changes in the protein expression of CAT at various stages of MASLD; while it revealed unchanged CAT expression in simple steatosis, a considerable reduction in fatty as well as non-fatty MASH liver samples was shown (Li et al. 2018). Indeed, downregulation of CAT protein expression was reported in MASH patients as compared to control (Shearn et al. 2017). In mouse models of MASLD, CAT protein expression was decreased by a HFD (Chen et al. 2015) as well as in a MSG model (Matouskova et al. 2015), and CAT protein levels were unchanged by a high-fat, high-fructose diet (An et al. 2021). Diminished protein CAT levels were observed also in oleic as well as palmitic acid-treated HepG2 cells (Chen et al. 2015; Sharma et al. 2020). In knockout mice deficient in hepatic S-adenosylmethionine synthesis, another model of MASH, a proteomic analysis showed upregulation in liver CAT expression as well as in the expression of SOD1 and GPx, a result which may reflect an adaptive mechanism to dissipate oxidative stress generated by oxidant genes (Santamaria et al. 2003).

Regarding CAT mRNA expression, an increase in mRNA levels was described in adults with simple steatosis (Aljomah et al. 2015; Ashla et al. 2010; Kohjima et al. 2007), adults with MASH (Aljomah et al. 2015; Baker et al. 2010) as well as in pediatric MASH patients (Desai et al. 2014; Moya et al. 2015) as compared to the levels in the normal liver, respectively. Interestingly, CAT mRNA levels did not differ among simple steatosis and MASH hepatic samples (Aljomah et al. 2015; Ashla et al. 2010). Increased hepatic mRNA expression of CAT was also reported in different diet-induced MASLD animal models (Jarukamjorn et al. 2016; Zhu et al. 2017).

Contrarily, Sreekumar et al. reported significantly downregulated CAT mRNA expression in the liver of patients with cirrhosis secondary to MASH (Sreekumar et al. 2003); while, conversely, no changes were observed in conventional MASH patients as compared to controls (Nagaya et al. 2015). Various animal models of MASLD showed downregulation in relative CAT mRNA expression in the liver (Nayan et al. 2021; Park et al. 2016; Pereira et al. 2017; Santos-López et al. 2016; Xia et al. 2016, 2018). Mitochondrial expression of CAT was also decreased in MASH mice as compared to their control counterparts (Krishnan et al. 2017). The expression of CAT was downregulated in HepG2 cells treated with fatty acids as compared to controls (Jain et al. 2018; Xia et al. 2018). On the other hand, no changes in mRNA CAT expression were found in MASLD male mice (Du et al. 2016; Gasparin et al. 2018; Rosas-Campos et al. 2024), rats (Tanaka et al. 2014), or MSG mice (Matouskova et al. 2015) as compared to respective controls.

### MASLD and glutathione peroxidases

Harmful H_2_O_2_ can also be eliminated by GPxs. These enzymes catalyze the reduction of H_2_O_2_ or organic hydroperoxides, forming water and corresponding alcohols, respectively. These enzymes have a higher affinity for H_2_O_2_ than CAT, and mainly use cellular glutathione (GSH) as a reductant (Asghari et al. 2020; Matoušková et al. 2018; Shin et al. 2018). Moreover, GSH serves not only as a reductant for GPxs, but also as a co-substrate for other antioxidant enzymes such as glutathione-S-transferases (GSTs) (Nobili et al. 2005). Eight GPxs (GPx1-GPx8) differing in structure, localization, function, and selenium dependence have been identified so far (Brigelius-Flohe and Maiorino 2013).

Huang et al. reported that the major functional genetic variation of GPx1 is a 593C > T polymorphism (rs1050450) in which its enzymatic activity is reduced. It has been also revealed that the GPx1 T/T genotype appeared in a significantly higher frequency in MASLD cases as compared to healthy controls (Huang et al. 2021). Furthermore, GPx1 variants Pro198Leu and C594T, which result in decreased enzymatic activity, were associated with increased risk of MASLD onset (Zhang et al. 2015, 2016).

Many inconsistent results have been reported regarding the mRNA/protein expression and/or activity of GPxs in association with MASLD or MASH (Table S3). Some studies reported higher GPx activity in the liver or erythrocytes of MASLD patients in comparison to healthy volunteers (Kumar et al. 2013; Perlemuter et al. 2005); similarly, the serum activity of GPx was significantly higher in early as well as advanced MASLD patients as compared to controls (Świderska et al. 2019). Among hemodialysis patients, higher GPx activity was displayed in those suffering from MASLD in comparison to those without MASLD (Wu et al. 2018). Another study showed upregulation of GPx activity in peripheral mononuclear leukocytes in MASLD patients as compared to healthy controls (Garcia et al. 2022). In various diet-induced MASLD/MASH animal models, increased hepatic activity of GPx was observed as well (Barbosa et al. 2021; Jarukamjorn et al. 2016; Nosrati et al. 2010; Valenzuela et al. 2018).

Interestingly, significant reduction in blood GPx activity was observed in MASLD obese patients as compared to controls (Kanoni et al. 2021), although most studies describing a decrease in GPx activity come from work done with MASLD animal models. Some studies report a statistically significant decrease in the GPx activity of HFD-fed obese rodents as compared to their lean counterparts (Huang et al. 2018; Noeman et al. 2011); similarly, it was reported that ob/ob mice (García-Ruiz and Fernández-Checa 2018) as well as mice fed a high-fructose diet (Li and Lu 2018) exhibited a decrease in the GPx activity as compared to controls, respectively. A HFD caused a significant decrease in mitochondrial GPx1 activity in rats (Valdecantos et al. 2012). The serum as well as hepatic activity of GPx was reported to be reduced in various MASLD/MASH animal models as compared to their respective controls (Alfarisi et al. 2020; Dai et al. 2014; Dong et al. 2018; Gasparin et al. 2018; Kim et al. 2017; Li et al. 2021; Lu et al. 2019; Matouskova et al. 2015; Veeramani et al. 2017; Wasef et al. 2021; Yoshioka et al. 2010). Reduced GPx activity was also reported in HepG2 cells treated with a combination of fatty acids (Li et al. 2021; Su et al. 2018; Xia et al. 2018), as well as in mouse liver cell line NCTC1469 treated with palmitate (Chen et al. 2017), respectively.

No significant changes in GPx activity were observed in the erythrocytes of MASLD patients (Asghari et al. 2020; Perlemuter et al. 2005), serum of MASH adults (Koruk et al. 2004) nor in the liver of MASH adult (Videla et al. 2004) and MASH pediatric patients (Desai et al. 2014; Nobili et al. 2005) as compared to healthy control subjects, respectively. Indeed, no significant alterations in GPx activity were detected in rodents fed a high-fat/high-fructose diet as compared to rodents fed a standard diet (Carvalho et al. 2018; Jung and Kim 2013; Liu et al. 2020; Mendes et al. 2018; Song et al. 2017; Wat et al. 2016). Hepatic GPx activity in rats was observed to be elevated in females compared to males; however, no significant differences were detected between the control and MASLD groups within either sex (Ortenzi et al. 2024).

Regarding protein expression, hepatic protein expression of GPx1/2 was observed to be reduced in MASLD rats as compared to controls (Li et al. 2021). Protein expression of GPx1 and total GPx showed a significant reduction in HFD-fed mice compared to respective controls (Chen et al. 2015; Mendes et al. 2018). Moreover, a reduced protein level of GPx1 and GPx1/2 was also reported in HepG2 cells treated with palmitic and oleic acid, respectively (Chen et al. 2015; Li et al. 2021). Recent studies have shown that GPx4 protein level was significantly reduced in HepG2 cells treated with palmitic acid (Ye et al. 2023), whereas a marked increase in GPx4 protein expression was observed in the hepatic tissue of MASLD mice (Jakubek et al. 2024).

Regarding mRNA expression, gene expression of GPx1 was reported to display an increase in the liver of AMLN as well as HFD-fed mice (Boland et al. 2018; Du et al. 2016); in addition, mRNA expression of GPx7 was significantly upregulated in mice suffering from MASH fibrosis as compared to healthy controls as well as mice with simple steatosis (Kim et al. 2020). The elevated hepatic mRNA level of GPx was reported in mice fed a high-fat and high-fructose diet as compared to their healthy counterparts (Jarukamjorn et al. 2016). GPx2 mRNA expression was upregulated in MSG obese mice (Matouskova et al. 2015).

In patients with cirrhosis secondary to MASH, hepatic gene expression of GPx was reported to be significantly diminished as compared to healthy controls (Sreekumar et al. 2003). Downregulated mRNA expression of GPx was reported in white adipose tissue, while GPx mRNA expression was slightly yet significantly upregulated in the liver of KK-A^y^ mice as compared to C57BL/6 mice (Furukawa et al. 2004). In HFD-fed mice, GPx3 mRNA expression was decreased as compared to mice fed a standard diet (Xia et al. 2016, 2018). In addition, studies with other diet-induced MASLD animal models showed downregulation of GPx mRNA expression (Li et al. 2014; Mendes et al. 2018; Nayan et al. 2021; Park et al. 2016). Experiments with the HepG2 cell line treated with fatty acids showed a decrease in GPx mRNA expression (Jain et al. 2018; Xia et al. 2018).

Conversely, no changes in GPx1 mRNA expression were observed in MASH pediatric and adult patients (Desai et al. 2014; Nagaya et al. 2015) nor in a MASLD mice model (Gasparin et al. 2018) as compared to controls, respectively.

### MASLD and glutathione reductase

Organisms that use GSH for redox homoeostasis are not only able to synthesize GSH but are also characterized by their ability to recycle it. Glutathione reductase (GR) is an essential antioxidant homodimeric enzyme responsible for the conversion of oxidized glutathione (GSSG) into GSH through a process employing a nicotinamide adenine dinucleotide phosphate (NADPH) as a source of reducing equivalents. GSH is one of the most abundant reducing thiols present in the majority of cells, thus the GSSG conversion is very important reaction for maintaining high levels of GSH within the cell. The impairment of the balance between GSH and GSSG usually induces loss of ability to control oxidative stress (Couto et al. 2016; Nobili et al. 2005; Venancio-Brochi et al. 2021).

Studies investigating the relationship between mRNA/protein expression and/or activity of GR and MASLD/MASH vary in their conclusions (Table S4). Serum activity of GR was reported to be significantly higher in early as well as advanced MASLD patients as compared to a control group (Świderska et al. 2019). Significant elevation in the GR activity was also reported in HFD-fed mice (de Freitas Carvalho et al. 2019), mice fed a high-fat and high-cholesterol diet (Liu et al. 2020), as well as rats fed an iron-rich diet (Valenzuela et al. 2018) as compared to their respective control counterparts.

On the other hand, a study by Garcia et al. showed a reduction in GR activity in the peripheral mononuclear leukocytes of MASLD patients as compared to healthy controls (Garcia et al. 2022). A significant reduction in hepatic GR activity was observed in many MASLD/MASH rat models as compared to control groups (Carmiel-Haggai et al. 2005; Hanafi et al. 2018; Li et al. 2021; Thomàs-Moyà et al. 2008; Tsai et al. 2016; Zakaria et al. 2021). Recent study has also reported a significant decrease in GR activity in whole liver tissue, cytosol, and mitochondria of rats fed a high cholesterol and sodium cholate diet compared to the control group (Silja et al. 2023). Likewise, hyperlipidemic mice as well as mice fed a HFD displayed a significant decrease in hepatic GR activity in comparison to their healthy counterparts (Kim et al. 2017; Lee and Lee 2021; Valenzuela et al. 2017). Similar results were also obtained in HepG2 cells treated with oleic acid (Li et al. 2021).

On the other hand, some studies have reported no significant differences in GR activity in MASH children (Desai et al. 2014; Nobili et al. 2005) nor in MASLD patients as compared to healthy subjects (Kumar et al. 2013; Ma et al. 2020). In addition, some MASLD rodent models displayed no significant changes in GR activity as compared to control groups (Carvalho et al. 2018; Chaves Cayuela et al. 2020; Matouskova et al. 2015).

Transcriptomic analysis of the liver of pediatric patients revealed no changes in GR mRNA expression as compared to the liver of healthy controls (Desai et al. 2014). On the other hand, mRNA expression of GR was reported to be downregulated in the liver of dietary MASLD rat models (Nayan et al. 2021; Santos-López et al. 2016). mRNA as well as the protein expression of GR were reported to be significantly decreased in the liver of a MASLD mouse model as compared to a control group (Mendes et al. 2018), while no changes were detected in mRNA and protein expression of GR in MSG mice (Matouskova et al. 2015). Recently, it has been revealed that female mice fed a methionine/choline-deficient diet showed no significant changes in hepatic mRNA expression of GR compared to controls (Rodriguez-Ramiro et al. 2024). However, HepG2 cells exposed to a combination of fatty acids exhibited a marked increase in GR gene expression (Longhitano et al. 2024).

### MASLD and glutathione S-transferases

Glutathione-S-transferases (GSTs) play a crucial role in phase II detoxification, as these isoenzymes protect cells against endogenous oxidative stress and exogenous toxins. The key reaction catalyzed by GSTs is the conjugation of electrophilic xenobiotics with the endogenous tripeptide GSH. GSTs are divided according to cellular localization into cytosolic, microsomal, and mitochondrial families. Recently, seven classes of mammalian cytosolic isoforms of GST enzymes have been recognized (alpha (A), mu (M), pi (P), sigma (S), zeta (Z), theta (T), and omega (O)). Among these, alpha-class GSTs (GSTA) have been shown to be the predominant isoenzymes in hepatocytes (Boušová and Skálová 2012; Hardwick et al. 2010; Prysyazhnyuk et al. 2021). Because GSH is a cofactor for GST isoforms, disruptions in GSH synthesis have the potential to severely disturb the detoxifying capacity of hepatic GSTs (Hardwick et al. 2010).

Regarding gene polymorphisms, carrying of the G allele of GSTP1 A313G polymorphism is considered to be a risk factor for MASLD development in humans. Moreover, no differences in the prevalence of the GSTT1 nor GSTM1 null genotype were observed between MASLD patients and healthy controls. Interestingly, deletion of GSTT1 and GSTM1 genes in MASLD patients was associated with lower GSH content (Prysyazhnyuk et al. 2021). Similarly, a study conducted by Oniki et al. reported that carrying of the GSTM1 null genotype, the GSTP1 A/B or B/B genotype, and the GSTA1 A/B or B/B genotype is associated with higher risk for MASLD development, as these genotypes show lower substrate affinity or lower hepatic expression compared to wild-type genotypes (Oniki et al. 2013).

Many inconsistent results regarding changes in the mRNA/protein expression and/or activity of GSTs in association with MASLD/MASH have been published (Table S5). It was reported that MASH children displayed higher GST activity compared to healthy subjects (Nobili et al. 2005). Increased hepatic GST activity was also reported in some MASLD animal models as compared to control groups (Ali et al. 2016; Lu et al. 2019). Contrarily, activity of GST was reported to be reduced in fatty MASH patients compared to healthy individuals (Shearn et al. 2017). Furthermore, a decrease in total GST activity advanced with MASLD progression in humans (Hardwick et al. 2010). In numerous animal MASLD models, a decrease in GST specific activity was observed (Hanafi et al. 2018; Chenna et al. 2024; Noeman et al. 2011; Zakaria et al. 2021). Interestingly, GST specific activity was increased in the kidney, while it was reduced in the liver and heart of male MSG mice (Bousova et al. 2017; Matouskova et al. 2015). Surprisingly, no significant changes in GST activity were observed in some dietary induced MASLD mouse models as compared to controls (Chaves Cayuela et al. 2020; Liu et al. 2020).

The protein expression of individual GST isoforms may follow a different trend in MASLD progression. For example, GSTA and GSTP protein expression tended to increase, whereas GSTM protein levels decreased with the progression of the disease (Hardwick et al. 2010). Indeed, GSTP protein expression was reported to be elevated in the liver of both fatty and non-fatty MASH patients as compared to healthy controls (Shearn et al. 2017). Similarly, hepatic GSTP protein expression was upregulated in rats on a choline-deficient/L-amino acid-defined diet (Endo et al. 2013). In the liver of MSG mice, expression of GSTM and GSTA was upregulated, while GSTP expression was downregulated (Matouskova et al. 2015). Conversely, a decrease or no change in GST protein levels were observed in rats fed a high-fat or high-fructose diet (Cai et al. 2014; Wang et al. 2018; Zhao et al. 2018b). Recent study has also demonstrated a significant decrease in the protein expression of GSTA1 in the liver of HFD-fed mice (Jiang et al. 2024). As MASLD and hypertension are common co-morbidities, a study regarding differences in GSTM1 and GSTO1 protein expression in normotensive and hypertensive rats was performed which showed significant GSTO1 downregulation and no change in GSTM1 levels in hypertensive rats (Svoboda and Kawaja 2012). In HepG2 cells, treatment with oleic acid caused an elevation in GST protein levels (Zhang et al. 2018b), while fructose-treated cells showed reduced GST (Zhao et al. 2018b).

Regarding gene expression, MASLD and MASH patients displayed elevated hepatic mRNA levels of GSTA1 as compared to a healthy group (Lee et al. 2016). Accordingly, mRNA levels of the GST isoforms A1, A2, A4, M3, and P1 tended to increase with MASLD progression in human subjects (Hardwick et al. 2010). On the other hand, it was reported that earlier stages of MASLD are accompanied by a downregulation of GSTM, a downregulation also prominent in MASH patients (Younossi et al. 2005). Tissue-specific differences in the MASLD-induced effect on GST mRNA/protein expression were observed in MSG male mice, in which renal GSTA1/2 and hepatic GSTM3 expressions were upregulated, while expression of GSTP1/2 was downregulated in the liver and heart (Bousova et al. 2017; Matouskova et al. 2015). Recently, a significant decrease in hepatic GSTA3 mRNA expression of female mice fed a methionine/choline-deficient diet compared to controls was reported, while no changes in GSTM2 expression were observed (Rodriguez-Ramiro et al. 2024). Increased mRNA levels of GSTA1 and GSTA2 were reported in a mouse model of MASH as well as in HepG2 cells treated with oleic acid, respectively (Lee et al. 2016; Li et al. 2020). On the other hand, a significant decrease in GST mRNA levels were reported in MASH rat models (Cai et al. 2014; Wang et al. 2018). Surprisingly, no changes were reported in GSTA3, GSTA5, and GSTM1 mRNA levels (Yu et al. 2021), nor in total GST mRNA (Stefano et al. 2015) in MASLD rat models as compared to control groups, respectively. Similarly, no changes in GSTA2 gene expression were observed in a MASLD mouse model (Ke et al. 2022).

### MASLD and NAD(P)H:quinone oxidoreductase 1

NAD(P)H:quinone oxidoreductase 1 (NQO1) has attracted interest due to its role in cell defense. NQO1 is a flavoenzyme responsible for the two-electron reduction of a wide range of quinones to stable hydroquinones. This detoxification process prevents the formation of the toxic semiquinone radicals and ROS, which are formed by one-electron reduction. The radicals and ROS can further react with intracellular thiol groups to cause significant cell damage (Hardwick et al. 2010; Ross and Siegel 2021; Zhang et al. 2018a).

Although NQO1 is not as studied in the field of MASLD, the studies that have been conducted to date have shown varying results regarding the expression and/or activity of this enzyme in MASLD/MASH patients as well as in many MASLD/MASH animal or cell models (Table S6).

According to a study by Hardwick et al., the specific activity of NQO1 as well as mRNA and protein expression tended to increase with disease progression, with mRNA and protein expression differing greatly (Hardwick et al. 2010). The type of experimental diet influenced the specific activity of NQO1: while the hepatic NQO1 activity of rats fed a methionine/choline-deficient diet was significantly increased, no significant changes were observed in HFD-fed rats (Lickteig et al. 2007), and a high-fat diet in combination with low doses of streptozotocin also led to no changes as compared to normal diet-fed controls (Vornoli et al. 2014). In MSG mice, the specific activity of NQO1 in the liver was three times higher than in the control mice (Matouskova et al. 2015).

Regarding protein expression, protein levels of NQO1 were markedly increased in the liver of fatty as well as non-fatty MASH patients as compared to those with simple steatosis as well as to healthy controls (Hardwick et al. 2010). Similarly, significantly elevated NQO1 protein levels were shown in HFD-fed mice as well as in HepG2 cells treated with oleic acid as compared to respective controls (Zhang et al. 2018b). In MASLD rodent models, increase in NQO1 protein expression was reported (Endo et al. 2013; Matouskova et al. 2015; Wang et al. 2018). Conversely, mice liver with CCl_4_-induced fibrosis showed significantly decreased NQO1 protein expression with respect to controls (Zhu et al. 2021). Moreover, many other studies reported significant downregulation of hepatic NQO1 protein levels in various animal MASLD models (Dong et al. 2018; Jin et al. 2021; Pan et al. 2024; Xia et al. 2016; Ye et al. 2022; Zhao et al. 2018b). Still, no significant changes were reported in a number of rodent diet-induced MASLD models (Fan et al. 2017; Hou et al. 2016; Lickteig et al. 2007; Xie et al. 2020; Yu et al. 2021). In HepG2 cells, protein expression of NQO1 was either significantly decreased or remained unchanged upon treatment with oleic acid, a combination of fatty acids or fructose (Jin et al. 2021; Sharma et al. 2020; Zhao et al. 2018a, 2018b).

In MASLD patients, mRNA expression of NQO1 tended to increase with progression of the disease (Hardwick et al. 2010; Lee et al. 2016). Elevation in NQO1 expression in advanced MASLD patients as compared to those with the mild form of the disease was caused by the hypomethylation in the differently methylated regions (Hotta et al. 2018). Upregulation in hepatic NQO1 gene expression was found in various rodent diet-induced or MSG-induced MASLD models (Deng et al. 2019; Fisher et al. 2008; Lee et al. 2016; Matouskova et al. 2015; Wang et al. 2018; Zhu et al. 2017). Sex differences in NQO1 mRNA expression were observed in the liver of mice on a cafeteria diet. NQO1 mRNA expression was significantly upregulated only in female mice, while the increase was insignificant in male mice (Gasparin et al. 2018). Induction in NQO1 mRNA was also reported in HepG2 cells treated with oleic acid (Li et al. 2020) or a combination of oleic and palmitic acid (Longhitano et al. 2024).

Conversely, a study by Zhao et al. showed significantly decreased mRNA levels, but no change in protein levels of NQO1 in HepG2 cells treated with a combination of fatty acids (Zhao et al. 2018a). In various diet-induced animal models of MASLD, downregulation of hepatic NQO1 mRNA levels was reported (Ke et al. 2022; Xu et al. 2021; Zilu et al. 2019), while no significant changes were found in several other studies (Yu et al. 2021; Zheng et al. 2021).

### MASLD and glutathione level

One of the most important cellular antioxidants, GSH contributes to the control of redox homoeostasis, as it acts as the major redox buffer in most cells. If a ROS donates one electron to a GSH molecule, the oxidized dimer GSSG is formed (Couto et al. 2016). As GSH is the co-substrate for many antioxidant enzymes, its sufficient level is important for proper function of the antioxidant defense system. Despite the many studies which have measured GSH levels in various models of MASLD/MASH, results have been very inconsistent (Table S7).

Significantly increased serum levels of GSH were reported in MASLD/MASH patients as compared to healthy volunteers (Koruk et al. 2004; Świderska et al. 2019). Substantial increase in hepatic and erythrocytic GSH levels (Janevski et al. 2011), as well as in total GSH level (Barbosa et al. 2021; de Freitas Carvalho et al. 2019) was reported in diet-induced MASLD/MASH rodent models.

Nevertheless, numerous studies have shown reduced GSH levels in association with MASLD/MASH. Patients with MASLD/MASH had significantly lower GSH levels in comparison to healthy volunteers (Kumar et al. 2013; Lickteig et al. 2007; Malaguarnera et al. 2005). GSH content was reported to be reduced by 57% and 27% in patients with steatosis and in patients with steatohepatitis as compared to healthy controls, respectively (Videla et al. 2004). Moreover, GSH content was markedly reduced in the circulating monocytes, CD^4+^ and CD^8+^ T-lymphocytes of MASLD patients as compared to healthy controls (Garcia et al. 2022). Reduced hepatic GSH levels were reported in numerous diet-induced MASLD/MASH rat and mice models (Dai et al. 2014; Gasparin et al. 2018; Hanafi et al. 2018; Chenna et al. 2024; Korish and Arafah 2013; Krishnan et al. 2017; Nayan et al. 2021; Pacana et al. 2015; Valenzuela et al. 2018; Veeramani et al. 2017; Ye et al. 2023; Yoshioka et al. 2010). Krishnan et al. reported decreased mitochondrial GSH level in mice on a fast-food diet compared to mice on a standard diet (Krishnan et al. 2017). Sex differences in hepatic GSH levels were observed in MASLD mice; a significant decrease was found in female mice, whereas no change was detected in male mice (Gasparin et al. 2018). The content of GSH was diminished in NCTC1469 cells treated with palmitate (Chen et al. 2017) as well as in HepG2 cells treated with fatty acids (Chen et al. 2015; Li et al. 2021; Longhitano et al. 2024).

In adult MASLD/MASH patients, the GSH/GSSG redox ratio seemed to decrease with disease severity (Hardwick et al. 2010). In the study by Nobili et al., total GSH level was comparable between MASH and healthy children, although the mean value of the level of GSSG was increased in the MASH group as compared to healthy controls (Nobili et al. 2005). Additionally, no changes in the GSH content were observed in MASLD animal models (Chen et al. 2022; Vornoli et al. 2014; Xia et al. 2018; Xu et al. 2017). Several studies reported a substantial reduction in relative liver GSH/GSSG ratio in diet-induced MASLD rat models (Hou et al. 2016; Valdecantos et al. 2012; Zhao et al. 2018a) or genetically-induced MASLD mice (Zhang et al. 2024), but no change in total GSH level as compared to a control group (Carvalho et al. 2018).

## Vitamin E and its impact on antioxidant enzymes in MASLD/MASH

Vitamin E, a crucial lipid-soluble antioxidant, plays a significant role in mitigating oxidative stress and retarding the pathogenesis of MASH by donating a hydrogen ion from its chromanol ring to scavenge lipid peroxyl radicals (Perumpail et al. 2018). The naturally occurring forms of vitamin E encompass eight variants, distinguished by the positions and numbers of methylation patterns on the chromanol ring, resulting in four isomers identified as alpha (α), beta (β), delta (δ), and gamma (γ) tocopherols and tocotrienols (Nor Azman et al. 2018; Pacana and Sanyal 2012). Tocotrienols are characterized by a chromanol ring attached to an unsaturated isoprenoid side chain, while tocopherols contain a chromanol ring attached to a saturated alkyl side chain. The presence of three chiral centers in the saturated side chain, presenting as either R- or S-configuration, leads to eight different stereoisomers, among which only α-tocopherol in its RRR conformation is selectively retained and incorporated into lipoproteins, while non-α-tocopherol congeners are preferentially metabolized and excreted (Podszun and Frank 2021). Thus, supplementation with α-tocopherol may enhance the activity and concentrations of endogenous antioxidants in the liver.

Studies have demonstrated that tocotrienol supplementation at a dosage of 150 mg/day notably elevated erythrocytic SOD and GPx activity in healthy older females after 6 months of treatment compared to the control group. Similarly, supplementation with α-tocopherol at a dosage of 400 IU/day resulted in a significant increase in the GSH/GSSG ratio in these subjects (Nor Azman et al. 2018).

In mice subjected to a methionine-choline-deficient diet, vitamin E antioxidant therapy restored GSH levels (Phung et al. 2009) and markedly increased hepatic SOD activity (Nan et al. 2009). Rats fed a HFD along with a dose of 30 IU/kg/day of vitamin E showed significantly enhanced hepatic SOD and GPx activities compared to respective control group (Bai et al. 2023). Conversely, intraperitoneal administration of vitamin E at a dose of 500 mg/kg to diabetic rats resulted in a non-significant increase in SOD activity, while GSH levels exhibited non-significant decrease in both the blood and liver tissue. GPx activity showed a significant decrease in the blood but a non-significant increase in the liver of diabetic rats supplemented with vitamin E compared to diabetic rats without supplementation (Seven et al. 2004). Additionally, no significant differences were observed in the hepatic GSH/GSSG ratio in guinea pigs fed a HFD supplemented with 250 mg/kg of RRR-α-tocopherol (Podszun et al. 2014). Some studies have suggested utilizing vitamin E in combination with other therapeutic agents, particularly with vitamin C, due to their synergistic or additive therapeutic effects. In its antioxidant function, vitamin E is primarily oxidized to the tocopheroxyl radical and then restored to tocopherol by vitamin C. Although diabetic rats exhibited lower activity of heart SOD and heart and liver CAT compared to control rats, supplementation with a combination of vitamins A, E, and C significantly enhanced the activity of only heart CAT (Tabei et al. 2015).

Current guidelines recommend using the RRR-α-tocopherol form of vitamin E for MASH treatment, given its antioxidant, anti-inflammatory, and anti-apoptotic properties. This, along with its favorable clinical profile, makes vitamin E a therapeutic option for non-diabetic patients with histologic evidence of MASH, particularly when dietary and lifestyle modifications fail to yield benefits (Perumpail et al. 2018). However, the existing data on the efficacy and safety of vitamin E is deemed insufficient to extend this recommendation to diabetic patients with MASH. Discrepant outcomes from earlier studies, largely attributable to small sample sizes and variations in primary endpoints and vitamin E formulations, have contributed to this cautious stance.

## Translation of the findings from MASLD/MASH animal models to humans

Animal models are crucial for better understanding human MASLD pathogenesis, identifying possible therapeutic targets and disease biomarkers, as well as testing novel drugs. However, the heterogeneity in MASLD etiology has made it challenging to develop a preclinical animal model that accurately reflects MASLD progression in humans. Despite the availability of various animal MASLD/MASH models, the one that best recapitulates human disease is yet to be defined. This is due to the diversity of models used varying in species, strains, genetic background, sex, age, study design, duration, and especially the methods of disease induction (Chua et al. 2022; Im et al. 2021; Nevzorova et al. 2020; Vacca et al. 2024). Moreover, the microbiome of animals grown in laboratory conditions and nature may vary greatly, affecting the potential response to drug therapies (Ramos et al. 2022).

The commonly used MASLD models include dietary, genetic, chemical, and combined approaches. While each model replicates certain aspects of the disease, none fully captures human MASLD pathology. Dietary-induced models, which mimic obesity and insulin resistance through high-fat, high-carbohydrate, or nutrient-deficient diets, often fail to reproduce the inflammation and fibrosis typical of advanced human MASLD. Advanced fibrosis is in these models usually achieved when combining nutritional intervention with a second proinflammatory hit. Among often used dietary animal models belong HFD, modified HFD (addition of carbohydrates, cholesterol, trans-fats or CCl_4_), methionine- and choline-deficient diet, and choline-deficient L-amino-defined diet. Nutrient-deficient diets does not replicate features of metabolic syndrome and weight gain typical for MASLD; their translatability is poor (Chua et al. 2022; Nevzorova et al. 2020; Vacca et al. 2024). The severity of HFD-induced MASLD may be dependent on the species, gender, strain, diet composition, and duration of feeding, moreover, high inter-individual variability in MASLD features is often observed. The enrichment of HFD with fructose induces development of metabolic syndrome, MASH, and fibrosis in experimental animals (Chua et al. 2022; Nevzorova et al. 2020). Prolonged feeding (> 38 weeks) with Western diets (combination of HFD with fructose and cholesterol) leads to the development of obesity, impaired glucose tolerance, and liver histopathological changes like those observed in MASH patients. This model shows good clinical translatability and aligns well with human MASH (Hansen et al. 2020; Vacca et al. 2024). Combination of Western diet with CCl_4_ administration resulted in rapid development of liver steatosis, inflammation, hepatocellular ballooning, and severe fibrosis, moreover, transcriptomic analysis showed that dysregulated molecular pathways were comparable to those of human MASH (Tsuchida et al. 2018). However, CCl_4_-derived metabolites have unknown role in human MASH (Nevzorova et al. 2020). Genetic models are useful for exploring genetic contributors to MASLD but rarely progress to severe inflammation or fibrosis without additional stressors. Chemical models, using agents like CCl_4_, diethylnitrosamine, thioacetamide or monosodium glutamate, induce liver fibrosis but do not fully represent the metabolic dysfunctions seen in human MASLD. Combined models attempt to replicate the full disease spectrum, incorporating steatosis, fibrosis, and metabolic features, but they remain incomplete as well (Chua et al. 2022).

Taken together, physiological differences between humans and animals, as well as variations in model parameters, contribute to inconsistencies in study outcomes. Increasing standardization and replication across animal models may help to address these issues.

One of the approaches to overcome the hindrances mentioned above is the development of clinically relevant in vitro human-based MASLD models. Various immortalized human cells lines (e.g. HepG2, HepaRG, LX-2), primary cell cultures (e.g. primary human hepatocytes, primary hepatic stellate cells), and pluripotent stem cells cultured in monolayers are commonly used in the MASLD/MASH research. Such monolayers are mainly used to study transport, storage, and metabolism of excess lipids by hepatocytes. However, these models do not reflect the heterogeneity and complexity of MASLD. Liver spheroids, organoids, and microfluidic devices represent more advanced three-dimensional MASLD models. The spheroids and organoids, static 3D models, enable co-cultivation of several cell types (e.g. hepatocytes with non-parenchymal cells) and prolonged cultivation time, moreover, these models better reflect MASLD complexity. Their major limitation is a lack of control over nutrients and gas exchange and waste disposal, which was overcome in microfluidic liver-on-chip platforms enabling automated control over these factors. The major challenge of the in vitro systems yet to be solved is to cover the whole complexity of the MASLD pathogenesis including the crosstalk between the liver, adipose tissue, and gut (Ramos et al. 2022; Wang et al. 2022).

## Conclusions

Due to the multiple affected pathways, the pathogenesis of MASLD is not fully understood. According to the generally accepted multiple-hit theory, oxidative stress and changes in antioxidant systems play an important role in MASLD progression as the starting point of hepatic and extrahepatic damage. Therefore, the relationship between MASLD development/progression and the changes in antioxidant systems has been the subject of numerous various studies, although the results obtained have been quite inconsistent. The reason for the inconsistency is primarily the different models that have been used in the studies (patients, different animal species and strains, various cell models). The patient cohorts often widely differed in number of subjects, age, disease severity, and many other factors. To induce MASLD in animals, different diets/procedures have been used for different periods, and there were also great differences in sampling times and monitored parameters. It is obvious that the results concerning one enzyme may be totally different if mRNA expression, protein expression or activity is assayed, due to time shifts in the changes among these parameters.

Despite the huge inconsistency of the results, antioxidant enzymes are consistently considered an important player in MASLD development and progression. It can be generalized that at the beginning of MASL development, the expression/ activity of antioxidant enzymes generally increases to protect organisms against increased ROS production. In MASH patients/models, the expression/activity of several antioxidants generally decreases due to damage of hepatic and extrahepatic cells, which in turn worsens the damage.

It is likely that changes in the expression/activities of a particular antioxidant enzyme will induce changes in other antioxidant enzymes. In order to evaluate the consequences of causes and consequences, it would be necessary to monitor changes in all antioxidant enzymes in a suitable animal model of MASLD for a substantial length of time with frequent sampling, as well as to evaluate each enzyme both at the level of mRNA/protein expression and at the level of activities. It would also be interesting to address the epigenetic regulation of the expression of these enzymes during the development/progression of MASLD to determine how changes in the expression of these enzymes occur. The obtained information might then be useful in the design of interventions targeting the selected antioxidant enzyme(s). The data summarized in present review show antioxidant enzymes as possible interesting targets to limit MASLD progression.

## Supplementary Information

Below is the link to the electronic supplementary material.

Supplementary file1 (DOCX 611 KB)

## Data Availability

Not applicable.
