# Supplementary material for: Metabolic dysfunction-associated steatotic liver disease-induced changes in the antioxidant system: a review
Source: Arch Toxicol. 2024 Oct 23;99(1):1–22. doi: 10.1007/s00204-024-03889-x (PMC11748479; doi:10.1007/s00204-024-03889-x)
Supplement: Supplementary file 1 — Supplementary file1 (DOCX 611 KB) [file 204_2024_3889_MOESM1_ESM.docx]

**Archives of Toxicology**

**Metabolic dysfunction-associated steatotic liver disease-induced changes in the antioxidant system: a review**

Gabriela Svobodová, Martin Horní, Eva Velecká, and Iva Boušová*

Department of Biochemical Sciences, Faculty of Pharmacy in Hradec Králové, Charles University, 500 05 Hradec Králové, Czech Republic

***Corresponding author:** Iva Boušová, Ph.D.; Email: [bousova@faf.cuni.cz](mailto:bousova@faf.cuni.cz); ORCID: 0000-0003-2863-717X

**Table 1** MASLD-induced changes in the activity and/or expression of superoxide dismutase.

| **Model** | **Effect** | **Catalytic activity (reference)** | **Protein expression (reference)** | **mRNA expression (reference)** |
| --- | --- | --- | --- | --- |
| MASLD/MASH patients | ↑ | (Asghari et al. 2020; Monserrat-Mesquida et al. 2020; Perlemuter et al. 2005; Świderska et al. 2019) SOD1 | (Shearn et al. 2017) | (Hotta et al. 2018; Kohjima et al. 2007) |
|  | ↓ | (Kanoni et al. 2021; Koruk et al. 2004; Videla et al. 2004) |  | (Sreekumar et al. 2003) |
|  | ↔ | (Kumar et al. 2013; Nobili et al. 2005; Perlemuter et al. 2005) SOD2 |  | (Nagaya et al. 2015) |
| HFD mice | ↑ | (Chen et al. 2015; Marinho et al. 2018) |  |  |
|  | ↓ | (de Oliveira et al. 2015; Feng et al. 2017; Huang et al. 2018; Chen et al. 2015; Chen et al. 2017; Chen et al. 2022a; Ke et al. 2022; Kim et al. 2018; Murakami et al. 2018; Santos et al. 2018; Su et al. 2018; Valenzuela et al. 2017; Veeramani et al. 2017; Wu et al. 2015; Xia et al. 2016; Xia et al. 2018; Ye et al. 2022) liver | (Chen et al. 2015) | (Xia et al. 2016; Xia et al. 2018) |
|  | ↔ | (Mendes et al. 2018; Nunes-Souza et al. 2016; Su et al. 2018; Xu et al. 2017) serum |  | (Du et al. 2016) |
| HFD rats | ↑ | (Barbosa et al. 2021; Song et al. 2017; Tsai et al. 2016) |  |  |
|  | ↓ | (Amirinejad et al. 2021; Carmiel-Haggai et al. 2005; Deng et al. 2019; Hou et al. 2016; Li et al. 2021; Nayan et al. 2021; Shatoor et al. 2021; Thomàs-Moyà et al. 2008; Valdecantos et al. 2012; Wang et al. 2017; Zakaria et al. 2021; Zhao et al. 2018a) | (Li et al. 2021) | (Nayan et al. 2021) |
|  | ↔ |  |  | (Tanaka et al. 2014; Zhu et al. 2017) |
| HFD + STZ rats | ↔ | (Vornoli et al. 2014) |  |  |
| HCD rat/hamster/rabbit | ↓ | (Santos-López et al. 2016)/(Lu et al. 2019)/(Alfarisi et al. 2020) |  | (Santos-López et al. 2016) |
| HCcholateD rats | ↔ | (Silja et al. 2023) |  |  |
| HFHCD mice/rats | ↓ | (Jung and Kim 2013)/(Yang et al. 2016) |  |  |
| HFrcD mice/rats | ↓ | (Li and Lu 2018)/(Carvalho et al. 2018) |  |  |
| HFHFrcD mice | ↑ | (Jarukamjorn et al. 2016) |  | (Jarukamjorn et al. 2016) |
| HFHFrcSucD mice | ↓ |  |  | (Rosas-Campos et al. 2024) |
| FFCD mice | ↑ | (Boland et al. 2018) |  |  |
|  | ↓ |  |  | (Krishnan et al. 2017) |
| Cafeteria diet mice | ↔ | (Gasparin et al. 2018) |  | (Gasparin et al. 2018) |
| Western diet mice | ↑ |  | (Jakubek et al. 2024) SOD3 |  |
|  | ↓ |  | (Jakubek et al. 2024) SOD2 |  |
|  | ↔ |  | (Jakubek et al. 2024) SOD1 |  |
| MCDD mice/rats | ↓ | (Jorgačević et al. 2014)/(Nosrati et al. 2010; Yoshioka et al. 2010) |  | (Park et al. 2016; Rodriguez-Ramiro et al. 2024) |
| MSG mice | ↓ | (Omogbiya et al. 2021) |  |  |
|  | ↔ | (Matouskova et al. 2015) | (Matouskova et al. 2015) | (Matouskova et al. 2015) |
| db/db mice | ↓ |  | (Nam et al. 2023) |  |
| Iron-rich diet rats | ↑ | (Valenzuela et al. 2018) |  |  |
| LPS-induced liver injury mice | ↓ | (Dong et al. 2018) |  |  |
| CCl_4_-induced liver injury rats | ↓ | (Dai et al. 2014) |  |  |
| KK-A^y^ mice | ↓ |  |  | (Furukawa et al. 2004) |
| FFAs-treated HepG2 cell | ↓ | (Li et al. 2021; Li et al. 2020; Su et al. 2018; Xia et al. 2018) | (Chen et al. 2015; Sharma et al. 2020) | (Jain et al. 2018; Xia et al. 2018) |

HFD, high-fat diet; HCD, high-cholesterol diet; STZ, streptozotocin; HFHCD, high-fat high-cholesterol diet; HCcholateD rats, high cholesterol + sodium cholate diet; HFrcD, high-fructose diet; HFHFrcD, high-fat high-fructose diet; HFHFrcSucD, high fat high fructose and sucrose diet; FFCD, high-fat high-cholesterol high-fructose diet; MCDD, methionine- and choline-deficient diet; MSG, monosodium glutamate; LPS, lipopolysaccharide; FFA, free fatty acids

**Table 2** MASLD-induced changes in the activity and/or expression of catalase.

| **Model** | **Effect** | **Catalytic activity (reference)** | **Protein expression (reference)** | **mRNA expression (reference)** |
| --- | --- | --- | --- | --- |
| MASLD/MASH patients | ↑ | (Baker et al. 2010; Monserrat-Mesquida et al. 2020; Moya et al. 2015; Perlemuter et al. 2005) liver |  | (Aljomah et al. 2015; Ashla et al. 2010; Baker et al. 2010; Desai et al. 2014; Kohjima et al. 2007; Moya et al. 2015) |
|  | ↓ | (Das et al. 2008; Kumar et al. 2013; Świderska et al. 2019; Videla et al. 2004; Yesilova et al. 2005) | (Li et al. 2018; Shearn et al. 2017) | (Sreekumar et al. 2003) |
|  | ↔ | (Li and Lu 2018; Perlemuter et al. 2005) Ery | (Li et al. 2018) | (Nagaya et al. 2015) |
| HFD mice | ↓ | (de Oliveira et al. 2015; Feng et al. 2017; Chen et al. 2015; Kim et al. 2018; Marinho et al. 2018; Murakami et al. 2018; Santos et al. 2018; Su et al. 2018; Valenzuela et al. 2017; Veeramani et al. 2017; Xia et al. 2016; Xia et al. 2018; Zhang et al. 2018) | (Chen et al. 2015) | (Xia et al. 2016; Xia et al. 2018) |
|  | ↔ | (Mendes et al. 2018; Nunes-Souza et al. 2016; Wu et al. 2015) |  | (Du et al. 2016) |
| HFD rats | ↑ | (Barbosa et al. 2021) |  | (Zhu et al. 2017) |
|  | ↓ | (Carmiel-Haggai et al. 2005; Pereira et al. 2017; Sabir et al. 2022; Song et al. 2017; Thomàs-Moyà et al. 2008; Tsai et al. 2016; Zakaria et al. 2021) |  | (Nayan et al. 2021; Pereira et al. 2017) |
|  | ↔ | (Noeman et al. 2011) |  | (Tanaka et al. 2014) |
| HFD + STZ rats | ↔ | (Vornoli et al. 2014) |  |  |
| HCD rat | ↓ | (Santos-López et al. 2016) |  | (Santos-López et al. 2016) |
| HCCholateD rats | ↔ | (Silja et al. 2023) |  |  |
| HFHCD mice/rats | ↓ | (Korish and Arafah 2013) |  |  |
|  | ↔ | (Jung and Kim 2013) |  |  |
| HFrcD rats | ↑ | (Carvalho et al. 2018) |  |  |
| HFHFrcD mice | ↑ | (Jarukamjorn et al. 2016) |  | (Jarukamjorn et al. 2016) |
|  | ↔ |  | (An et al. 2021) |  |
| HFHFrcSucD mice | ↔ |  |  | (Rosas-Campos et al. 2024) |
| FFCD mice | ↑ | (Shin et al. 2018) |  |  |
|  | ↓ |  |  | (Krishnan et al. 2017) |
|  | ↔ | (Boland et al. 2018) |  |  |
| Cafeteria diet mice | ↓ | (Gasparin et al. 2018) |  |  |
|  | ↔ |  |  | (Gasparin et al. 2018) |
| Western diet mice | ↑ |  | (Jakubek et al. 2024) |  |
| MCDD mice/rats | ↓ | (Jorgačević et al. 2014)/(Yoshioka et al. 2010) |  | (Park et al. 2016) |
| MSG mice | ↓ | (Omogbiya et al. 2021) | (Matouskova et al. 2015) |  |
|  | ↔ | (Matouskova et al. 2015) |  | (Matouskova et al. 2015) |
| Iron-rich diet rats | ↑ | (Valenzuela et al. 2018) |  |  |
| CCl_4_-induced liver injury rats | ↓ | (Dai et al. 2014) |  |  |
| Ado-Met deficient mice | ↑ |  | (Santamaria et al. 2003) |  |
| FFAs-treated HepG2 cells | ↓ | (Su et al. 2018; Xia et al. 2018; Zhang et al. 2018) | (Chen et al. 2015; Sharma et al. 2020) | (Jain et al. 2018; Xia et al. 2018) |
| FFAs-treated rat hepatoma cells | ↑ | (Vecchione et al. 2016) |  |  |

Ado-Met, S-adenosylmethionine; HFHCD, high fat

**Table 3** MASLD-induced changes in the activity and/or expression of glutathione peroxidase.

| **Model** | **Effect** | **Catalytic activity (reference)** | **Protein expression (reference)** | **mRNA expression (reference)** |
| --- | --- | --- | --- | --- |
| MASLD/MASH patients | ↑ | (Garcia et al. 2022; Kumar et al. 2013; Perlemuter et al. 2005; Świderska et al. 2019; Wu et al. 2018) |  |  |
|  | ↓ | (Kanoni et al. 2021) |  | (Sreekumar et al. 2003) |
|  | ↔ | (Asghari et al. 2020; Desai et al. 2014; Koruk et al. 2004; Nobili et al. 2005; Perlemuter et al. 2005; Videla et al. 2004) |  | (Desai et al. 2014; Nagaya et al. 2015) |
| HFD mice | ↑ |  |  | (Du et al. 2016) |
|  | ↓ | (de Oliveira et al. 2015; Feng et al. 2017; Huang et al. 2018; Chen et al. 2015; Chen et al. 2017; Chen et al. 2022b; Lee and Lee 2021; Su et al. 2018; Valenzuela et al. 2017; Veeramani et al. 2017; Wu et al. 2015; Xia et al. 2016; Xia et al. 2018) | (Chen et al. 2015; Mendes et al. 2018) | (Xia et al. 2016; Xia et al. 2018) |
|  | ↔ | (Mendes et al. 2018) |  |  |
| HFD rats | ↑ | (Barbosa et al. 2021) |  |  |
|  | ↓ | (Amirinejad et al. 2021; Carmiel-Haggai et al. 2005; Hanafi et al. 2018; Li et al. 2021; Noeman et al. 2011; Thomàs-Moyà et al. 2008; Tsai et al. 2016; Valdecantos et al. 2012; Zakaria et al. 2021) | (Li et al. 2021) | (Nayan et al. 2021) |
|  | ↔ | (Ortenzi et al. 2024; Song et al. 2017; Wat et al. 2016) |  |  |
| HCD hamster/rabbit | ↓ | (Lu et al. 2019)/(Alfarisi et al. 2020) |  |  |
| HCcholateD rats | ↓ | (Silja et al. 2023) |  |  |
| HFHCD mice | ↔ | (Jung and Kim 2013; Liu et al. 2020) |  |  |
| HFrcD mice/rats | ↓ | (Li and Lu 2018) |  |  |
|  | ↔ | (Carvalho et al. 2018) |  |  |
| HFHFrcD mice | ↑ | (Jarukamjorn et al. 2016) |  |  |
| FFCD mice | ↑ |  |  | (Boland et al. 2018) |
| Cafeteria diet mice | ↓ | (Gasparin et al. 2018) |  |  |
|  | ↔ |  |  | (Gasparin et al. 2018) |
| Western diet mice | ↑ |  | (Jakubek et al. 2024) GPx4 |  |
| MCDD mice/rats | ↑ | (Ye et al. 2021)/(Nosrati et al. 2010) |  |  |
|  | ↓ | (Yoshioka et al. 2010) |  | (Li et al. 2014; Mendes et al. 2018; Park et al. 2016) |
| CDAHFD mice | ↑ |  |  | (Kim et al. 2020) |
| Ado-Met-deficient mice | ↑ |  | (Santamaria et al. 2003) |  |
| MSG mice | ↑ |  |  | (Matouskova et al. 2015) |
|  | ↓ | (Matouskova et al. 2015) |  |  |
| LPS-induced liver injury mice | ↓ | (Dong et al. 2018) |  |  |
| Iron-rich diet rats | ↑ | (Valenzuela et al. 2018) |  |  |
| CCl_4_-induced liver injury rats | ↓ | (Dai et al. 2014) |  |  |
| KK-A^y^ mice | ↓ |  |  | (Furukawa et al. 2004) |
| Ob/ob mice | ↓ | (García-Ruiz and Fernández-Checa 2018) |  |  |
| FFAs-treated HepG2 cells | ↑ |  |  | (Longhitano et al. 2024) |
|  | ↓ | (Li et al. 2021; Su et al. 2018; Xia et al. 2018) |  | (Jain et al. 2018; Xia et al. 2018) |
| PA-treated HepG2 cells | ↓ |  | (Chen et al. 2015; Li et al. 2021; Ye et al. 2023) |  |
| PA-treated NCTC1469 cells | ↓ | (Chen et al. 2017) |  |  |

CDAHFD, choline-deficient, L-amino acid-defined, high-fat diet; PA, palmitic acid

**Table 4** MASLD-induced changes in the activity and/or expression of glutathione reductase.

| **Model** | **Effect** | **Catalytic activity (reference)** | **Protein expression (reference)** | **mRNA expression (reference)** |
| --- | --- | --- | --- | --- |
| MASLD/MASH patients | ↑ | (Świderska et al. 2019) |  |  |
|  | ↓ | (Garcia et al. 2022) |  |  |
|  | ↔ | (Desai et al. 2014; Kumar et al. 2013; Ma et al. 2020; Nobili et al. 2005) |  | (Desai et al. 2014) |
| HFD mice | ↑ | (de Freitas Carvalho et al. 2019) |  |  |
|  | ↓ | (Lee and Lee 2021; Valenzuela et al. 2017) | (Mendes et al. 2018) | (Mendes et al. 2018) |
| HFD rats | ↓ | (Carmiel-Haggai et al. 2005; Hanafi et al. 2018; Li et al. 2021; Thomàs-Moyà et al. 2008; Tsai et al. 2016; Zakaria et al. 2021) |  | (Nayan et al. 2021) |
| HCD rats | ↓ |  |  | (Santos-López et al. 2016) |
| HCcholateD rats | ↓ | (Silja et al. 2023) |  |  |
| HFHCD mice | ↓ | (Liu et al. 2020) |  |  |
| HFrcD rats | ↔ | (Carvalho et al. 2018) |  |  |
| MCDD mice | ↔ | (Chaves Cayuela et al. 2020) |  | (Rodriguez-Ramiro et al. 2024) |
| MSG mice | ↔ | (Matouskova et al. 2015) | (Matouskova et al. 2015) | (Matouskova et al. 2015) |
| Iron-rich diet rats | ↑ | (Valenzuela et al. 2018) |  |  |
| FFAs-treated HepG2 cells | ↑ |  |  | (Longhitano et al. 2024) |
| OA-treated HepG2 cells | ↓ | (Li et al. 2021) |  |  |

OA, oleic acid

**Table 5** MASLD-induced changes in the activity and/or expression of glutathione S-transferase.

| **Model** | **Effect** | **Catalytic activity (reference)** | **Protein expression (reference)** | **mRNA expression (reference)** |
| --- | --- | --- | --- | --- |
| MASLD/MASH patients | ↑ | (Nobili et al. 2005) | (Hardwick et al. 2010; Shearn et al. 2017) GSTA | (Hardwick et al. 2010; Lee et al. 2016) |
|  | ↓ | (Hardwick et al. 2010; Shearn et al. 2017) | (Hardwick et al. 2010) GSTM | (Younossi et al. 2005) |
| HFD mice | ↑ |  | (Zhang et al. 2018) |  |
|  | ↓ | (Santos et al. 2018; Xu et al. 2017) | (Jiang et al. 2024) GSTA1 |  |
|  | ↔ |  |  | (Ke et al. 2022) |
| HFD rats | ↓ | (Hanafi et al. 2018; Noeman et al. 2011; Zakaria et al. 2021) | (Cai et al. 2014) | (Cai et al. 2014; Wang et al. 2018) |
|  | ↔ |  | (Wang et al. 2018) | (Yu et al. 2021) |
| HCD hamster | ↑ | (Lu et al. 2019) |  |  |
| HCcholateD rats | ↔ | (Silja et al. 2023) |  |  |
| HFHCD mice | ↔ | (Liu et al. 2020) |  |  |
| HFrcD rats | ↔ |  | (Zhao et al. 2018b) |  |
| MCDD mice/rats | ↑ | (Ali et al. 2016) |  | (Lee et al. 2016) |
|  | ↓ |  |  | (Rodriguez-Ramiro et al. 2024) GSTA3 |
|  | ↔ | (Chaves Cayuela et al. 2020) |  | (Rodriguez-Ramiro et al. 2024) GSTM2 |
| CDAAD rats | ↑ |  | (Endo et al. 2013) |  |
| CDHFD rats | ↔ |  |  | (Stefano et al. 2015) |
| MSG mice | ↑ |  | (Matouskova et al. 2015) GSTA, GSTM | (Matouskova et al. 2015) GSTM3 |
|  | ↓ | (Matouskova et al. 2015) | (Matouskova et al. 2015) GSTP | (Matouskova et al. 2015) GSTP1/2 |
| FFAs-treated HepG2 cells | ↑ |  |  | (Longhitano et al. 2024) GSTP1 |
|  | ↓ |  | (Jiang et al. 2024) GSTA1 |  |
| OA-treated HepG2 cells | ↑ |  | (Zhang et al. 2018) | (Li et al. 2020) |
|  | ↓ |  | (Jiang et al. 2024) GSTA1 |  |
| PAtreated HepG2 cells | ↓ |  | (Jiang et al. 2024) GSTA1 |  |
| Frc-treated HepG2 cells | ↓ |  | (Zhao et al. 2018b) |  |

CDHFD, choline-deficient, high-fat diet; CDAAD, choline-deficient/L-amino acid-defined diet; Frc, fructose

**Table 6** MASLD-induced changes in the activity and/or expression of NADPH:quinone oxidoreductase.

| **Model** | **Effect** | **Catalytic activity (reference)** | **Protein expression (reference)** | **mRNA expression (reference)** |
| --- | --- | --- | --- | --- |
| MASLD/MASH patients | ↑ | (Hardwick et al. 2010) | (Hardwick et al. 2010) | (Hardwick et al. 2010; Hotta et al. 2018; Lee et al. 2016) |
| HFD mice | ↑ |  | (Zhang et al. 2018) |  |
|  | ↓ |  | (Jin et al. 2021; Xia et al. 2016; Ye et al. 2022) | (Ke et al. 2022; Xu et al. 2021; Zilu et al. 2019) |
|  | ↔ |  |  | (Zheng et al. 2021) |
| HFD rats | ↑ |  | (Cai et al. 2014; Wang et al. 2018) | (Cai et al. 2014; Deng et al. 2019; Fan et al. 2017; Wang et al. 2018; Zhu et al. 2017) |
|  | ↔ | (Lickteig et al. 2007) | (Fan et al. 2017; Hou et al. 2016; Lickteig et al. 2007; Yu et al. 2021) | (Yu et al. 2021) |
| HFD + STZ rats | ↔ | (Vornoli et al. 2014) |  |  |
| HFrcD rats | ↓ |  | (Zhao et al. 2018b) |  |
| Cafeteria diet mice | ↑ |  |  | (Gasparin et al. 2018) |
| Western diet mice | ↔ |  | (Xie et al. 2020) |  |
| MCDD mice/rats | ↑ | (Lickteig et al. 2007) |  | (Fisher et al. 2008; Lee et al. 2016) |
|  | ↔ |  |  | (Rodriguez-Ramiro et al. 2024) |
| CDAAD rats | ↑ |  | (Endo et al. 2013) |  |
| MSG mice | ↑ | (Matouskova et al. 2015) | (Matouskova et al. 2015) | (Matouskova et al. 2015) |
| LPS-induced liver injury mice | ↓ |  | (Dong et al. 2018) |  |
| CCl_4_- induced liver injury mice | ↓ |  | (Zhu et al. 2021) |  |
| OA-treated HepG2 cells | ↑ |  | (Zhang et al. 2018) | (Li et al. 2020) |
|  |  |  |  |  |
|  | ↓ |  | (Sharma et al. 2020) |  |
| FFAs-treated HepG2 cells | ↑ |  |  | (Longhitano et al. 2024) |
|  | ↓ |  | (Jin et al. 2021) | (Zhao et al. 2018a) |
|  | ↔ |  | (Zhao et al. 2018a) |  |
| Frc-treated HepG2 cells | ↓ |  | (Zhao et al. 2018b) |  |

**Table 7** MASLD-induced changes in the level of reduced glutathione and oxidized glutathione.

| **Model** | **Effect** | **GSH level (reference)** | **GSSG level (reference)** | **GSH/GSSG ratio (reference)** |
| --- | --- | --- | --- | --- |
| MASLD/MASH patients | ↑ | (Koruk et al. 2004; Świderska et al. 2019) | (Nobili et al. 2005) |  |
|  | ↓ | (Kumar et al. 2013; Malaguarnera et al. 2005; Videla et al. 2004) | (Hardwick et al. 2010) | (Hardwick et al. 2010; Nobili et al. 2005) |
|  | ↔ | (Nobili et al. 2005) |  |  |
| HFD mice | ↑ | (de Freitas Carvalho et al. 2019) |  |  |
|  | ↓ | (Du et al. 2016; Chen et al. 2017; Ke et al. 2022; Kim et al. 2018; Murakami et al. 2018; Valenzuela et al. 2017; Veeramani et al. 2017; Xia et al. 2016) |  |  |
|  | ↔ | (Chen et al. 2022b; Xia et al. 2018; Xu et al. 2017) |  |  |
| HFD rats | ↑ | (Barbosa et al. 2021) |  |  |
|  | ↓ | (Carmiel-Haggai et al. 2005; Deng et al. 2019; Elshazly 2015; Hanafi et al. 2018; Li et al. 2021; Lickteig et al. 2007; Nayan et al. 2021; Noeman et al. 2011; Shatoor et al. 2021; Tsai et al. 2016; Zakaria et al. 2021) |  | (Hou et al. 2016; Valdecantos et al. 2012; Zhao et al. 2018a) |
| HFD + STZ rats | ↔ | (Vornoli et al. 2014) |  |  |
| HFHCD mice/rats | ↓ | (Liu et al. 2020; Pacana et al. 2015)/(Korish and Arafah 2013; Yang et al. 2016) |  |  |
| HCcholateD rats | ↓ | (Silja et al. 2023) |  |  |
| HFrcD rats | ↔ | (Carvalho et al. 2018) |  |  |
|  | ↓ |  |  | (Carvalho et al. 2018) |
| HFHFrcD mice | ↓ | (An et al. 2021; Jarukamjorn et al. 2016) |  |  |
| FFCD mice | ↓ | (Krishnan et al. 2017) |  |  |
| Cafeteria diet mice | ↓ | (Gasparin et al. 2018) female |  |  |
|  | ↔ | (Gasparin et al. 2018) male |  |  |
| MCDD mice/rats | ↓ | (Caballero et al. 2010; Jorgačević et al. 2014)/(Ali et al. 2016; Nosrati et al. 2010; Yoshioka et al. 2010) |  |  |
| MSG mice | ↓ | (Omogbiya et al. 2021) |  |  |
|  |  |  |  |  |
| db/db mice | ↑ |  |  | (Zhang et al. 2024) |
| Iron-rich diet rats | ↓ | (Valenzuela et al. 2018) |  |  |
| CCl_4_-induced liver injury rats | ↓ | (Dai et al. 2014) |  |  |
| FFAs-treated HepG2 cells | ↓ | (Chen et al. 2015; Li et al. 2021; Longhitano et al. 2024) |  |  |
| PA-treated NCTC1469 cells | ↓ | (Chen et al. 2017) |  |  |
| PA/OA-treated AML12 hepatocytes | ↑ |  |  | (Zhang et al. 2024) |

**References**

Alfarisi HAH, Ibrahim MB, Mohamed ZBH, Azahari N, Hamdan AHB, Che Mohamad CA (2020) Hepatoprotective Effects of a Novel Trihoney against Nonalcoholic Fatty Liver Disease: A Comparative Study with Atorvastatin. ScientificWorldJournal 2020:4503253 doi:10.1155/2020/4503253

Ali MH, Messiha BA, Abdel-Latif HA (2016) Protective effect of ursodeoxycholic acid, resveratrol, and N-acetylcysteine on nonalcoholic fatty liver disease in rats. Pharm Biol 54(7):1198-208 doi:10.3109/13880209.2015.1060247

Aljomah G, Baker SS, Liu W, et al. (2015) Induction of CYP2E1 in non-alcoholic fatty liver diseases. Exp Mol Pathol 99(3):677-81 doi:10.1016/j.yexmp.2015.11.008

Amirinejad A, Totmaj AS, Mardali F, et al. (2021) Administration of hydro-alcoholic extract of spinach improves oxidative stress and inflammation in high-fat diet-induced NAFLD rats. BMC Complement Med Ther 21(1):221 doi:10.1186/s12906-021-03396-x

An MY, Lee SR, Hwang HJ, Yoon JG, Lee HJ, Cho JA (2021) Antioxidant and Anti-Inflammatory Effects of Korean Black Ginseng Extract through ER Stress Pathway. Antioxidants (Basel) 10(1) doi:10.3390/antiox10010062

Asghari S, Hamedi-Shahraki S, Amirkhizi F (2020) Systemic redox imbalance in patients with nonalcoholic fatty liver disease. Eur J Clin Invest 50(4):e13211 doi:10.1111/eci.13211

Ashla AA, Hoshikawa Y, Tsuchiya H, et al. (2010) Genetic analysis of expression profile involved in retinoid metabolism in non-alcoholic fatty liver disease. Hepatol Res 40(6):594-604 doi:10.1111/j.1872-034X.2010.00646.x

Baker SS, Baker RD, Liu W, Nowak NJ, Zhu L (2010) Role of alcohol metabolism in non-alcoholic steatohepatitis. PLoS One 5(3):e9570 doi:10.1371/journal.pone.0009570

Barbosa PO, Souza MO, Silva MPS, et al. (2021) Açaí (Euterpe oleracea Martius) supplementation improves oxidative stress biomarkers in liver tissue of dams fed a high-fat diet and increases antioxidant enzymes' gene expression in offspring. Biomed Pharmacother 139:111627 doi:10.1016/j.biopha.2021.111627

Boland ML, Oldham S, Boland BB, et al. (2018) Nonalcoholic steatohepatitis severity is defined by a failure in compensatory antioxidant capacity in the setting of mitochondrial dysfunction. World J Gastroenterol 24(16):1748-1765 doi:10.3748/wjg.v24.i16.1748

Caballero F, Fernández A, Matías N, et al. (2010) Specific contribution of methionine and choline in nutritional nonalcoholic steatohepatitis: impact on mitochondrial S-adenosyl-L-methionine and glutathione. J Biol Chem 285(24):18528-36 doi:10.1074/jbc.M109.099333

Cai YQ, Zhang LZ, Wang DJ, et al. (2014) [Effect of Nrf2 and related factors on the progression of nonalcoholic steatohepatitis]. Zhongguo Ying Yong Sheng Li Xue Za Zhi 30(5):465-70

Carmiel-Haggai M, Cederbaum AI, Nieto N (2005) A high-fat diet leads to the progression of non-alcoholic fatty liver disease in obese rats. Faseb j 19(1):136-8 doi:10.1096/fj.04-2291fje

Carvalho MMF, Reis LLT, Lopes JMM, et al. (2018) Açai improves non-alcoholic fatty liver disease (NAFLD) induced by fructose. Nutr Hosp 35(2):318-325 doi:10.20960/nh.1294

Dai N, Zou Y, Zhu L, Wang HF, Dai MG (2014) Antioxidant properties of proanthocyanidins attenuate carbon tetrachloride (CCl4)-induced steatosis and liver injury in rats via CYP2E1 regulation. J Med Food 17(6):663-9 doi:10.1089/jmf.2013.2834

Das KS, Balakrishnan V, Mukherjee S, Vasudevan DM (2008) Evaluation of blood oxidative stress-related parameters in alcoholic liver disease and non-alcoholic fatty liver disease. Scand J Clin Lab Invest 68(4):323-34 doi:10.1080/00365510701673383

de Freitas Carvalho MM, Lage NN, de Souza Paulino AH, et al. (2019) Effects of açai on oxidative stress, ER stress, and inflammation-related parameters in mice with high fat diet-fed induced NAFLD. Sci Rep 9(1):8107 doi:10.1038/s41598-019-44563-y

de Oliveira PR, da Costa CA, de Bem GF, et al. (2015) Euterpe oleracea Mart.-Derived Polyphenols Protect Mice from Diet-Induced Obesity and Fatty Liver by Regulating Hepatic Lipogenesis and Cholesterol Excretion. PLoS One 10(12):e0143721 doi:10.1371/journal.pone.0143721

Deng Y, Tang K, Chen R, et al. (2019) Berberine attenuates hepatic oxidative stress in rats with non-alcoholic fatty liver disease via the Nrf2/ARE signalling pathway. Exp Ther Med 17(3):2091-2098 doi:10.3892/etm.2019.7208

Desai S, Baker SS, Liu W, et al. (2014) Paraoxonase 1 and oxidative stress in paediatric non-alcoholic steatohepatitis. Liver Int 34(1):110-7 doi:10.1111/liv.12308

Dong L, Han X, Tao X, et al. (2018) Protection by the Total Flavonoids from Rosa laevigata Michx Fruit against Lipopolysaccharide-Induced Liver Injury in Mice via Modulation of FXR Signaling. Foods 7(6) doi:10.3390/foods7060088

Du J, Zhang M, Lu J, et al. (2016) Osteocalcin improves nonalcoholic fatty liver disease in mice through activation of Nrf2 and inhibition of JNK. Endocrine 53(3):701-9 doi:10.1007/s12020-016-0926-5

Elshazly SM (2015) Ameliorative effect of nicorandil on high fat diet induced non-alcoholic fatty liver disease in rats. Eur J Pharmacol 748:123-32 doi:10.1016/j.ejphar.2014.12.017

Endo H, Niioka M, Kobayashi N, Tanaka M, Watanabe T (2013) Butyrate-producing probiotics reduce nonalcoholic fatty liver disease progression in rats: new insight into the probiotics for the gut-liver axis. PLoS One 8(5):e63388 doi:10.1371/journal.pone.0063388

Fan H, Ma X, Lin P, et al. (2017) Scutellarin Prevents Nonalcoholic Fatty Liver Disease (NAFLD) and Hyperlipidemia via PI3K/AKT-Dependent Activation of Nuclear Factor (Erythroid-Derived 2)-Like 2 (Nrf2) in Rats. Med Sci Monit 23:5599-5612 doi:10.12659/msm.907530

Feng X, Yu W, Li X, et al. (2017) Apigenin, a modulator of PPARγ, attenuates HFD-induced NAFLD by regulating hepatocyte lipid metabolism and oxidative stress via Nrf2 activation. Biochem Pharmacol 136:136-149 doi:10.1016/j.bcp.2017.04.014

Fisher CD, Jackson JP, Lickteig AJ, Augustine LM, Cherrington NJ (2008) Drug metabolizing enzyme induction pathways in experimental non-alcoholic steatohepatitis. Archives of toxicology 82(12):959-64 doi:10.1007/s00204-008-0312-z

Furukawa S, Fujita T, Shimabukuro M, et al. (2004) Increased oxidative stress in obesity and its impact on metabolic syndrome. J Clin Invest 114(12):1752-61 doi:10.1172/jci21625

García-Ruiz C, Fernández-Checa JC (2018) Mitochondrial Oxidative Stress and Antioxidants Balance in Fatty Liver Disease. Hepatol Commun 2(12):1425-1439 doi:10.1002/hep4.1271

Garcia CC, Piotrkowski B, Baz P, et al. (2022) A Decreased Response to Resistin in Mononuclear Leukocytes Contributes to Oxidative Stress in Nonalcoholic Fatty Liver Disease. Dig Dis Sci 67(7):3006-3016 doi:10.1007/s10620-021-07105-z

Gasparin FRS, Carreño FO, Mewes JM, et al. (2018) Sex differences in the development of hepatic steatosis in cafeteria diet-induced obesity in young mice. Biochim Biophys Acta Mol Basis Dis 1864(7):2495-2509 doi:10.1016/j.bbadis.2018.04.004

Hanafi MY, Zaher ELM, El-Adely SEM, et al. (2018) The therapeutic effects of bee venom on some metabolic and antioxidant parameters associated with HFD-induced non-alcoholic fatty liver in rats. Exp Ther Med 15(6):5091-5099 doi:10.3892/etm.2018.6028

Hardwick RN, Fisher CD, Canet MJ, Lake AD, Cherrington NJ (2010) Diversity in antioxidant response enzymes in progressive stages of human nonalcoholic fatty liver disease. Drug Metab Dispos 38(12):2293-301 doi:10.1124/dmd.110.035006

Hotta K, Kitamoto T, Kitamoto A, et al. (2018) Identification of the genomic region under epigenetic regulation during non-alcoholic fatty liver disease progression. Hepatol Res 48(3):E320-e334 doi:10.1111/hepr.12992

Hou C, Wang Y, Zhu E, et al. (2016) Coral calcium hydride prevents hepatic steatosis in high fat diet-induced obese rats: A potent mitochondrial nutrient and phase II enzyme inducer. Biochem Pharmacol 103:85-97 doi:10.1016/j.bcp.2015.12.020

Huang F, Wang J, Yu F, et al. (2018) Protective Effect of Meretrix meretrix Oligopeptides on High-Fat-Diet-Induced Non-Alcoholic Fatty Liver Disease in Mice. Mar Drugs 16(2) doi:10.3390/md16020039

Chaves Cayuela N, Kiyomi Koike M, Jacysyn JF, et al. (2020) N-Acetylcysteine Reduced Ischemia and Reperfusion Damage Associated with Steatohepatitis in Mice. International journal of molecular sciences 21(11) doi:10.3390/ijms21114106

Chen G, Xu R, Zhang S, et al. (2015) CYP2J2 overexpression attenuates nonalcoholic fatty liver disease induced by high-fat diet in mice. Am J Physiol Endocrinol Metab 308(2):E97-e110 doi:10.1152/ajpendo.00366.2014

Chen J, Liu J, Wang Y, et al. (2017) Wogonin mitigates nonalcoholic fatty liver disease via enhancing PPARα/AdipoR2, in vivo and in vitro. Biomed Pharmacother 91:621-631 doi:10.1016/j.biopha.2017.04.125

Chen JYS, Chua D, Lim CO, Ho WX, Tan NS (2022a) Lessons on Drug Development: A Literature Review of Challenges Faced in Nonalcoholic Fatty Liver Disease (NAFLD) Clinical Trials. International journal of molecular sciences 24(1) doi:10.3390/ijms24010158

Chen YM, Lian CF, Sun QW, et al. (2022b) Ramulus Mori (Sangzhi) Alkaloids Alleviate High-Fat Diet-Induced Obesity and Nonalcoholic Fatty Liver Disease in Mice. Antioxidants (Basel) 11(5) doi:10.3390/antiox11050905

Jain MR, Giri SR, Bhoi B, et al. (2018) Dual PPARα/γ agonist saroglitazar improves liver histopathology and biochemistry in experimental NASH models. Liver Int 38(6):1084-1094 doi:10.1111/liv.13634

Jakubek P, Kalinowski P, Karkucinska-Wieckowska A, et al. (2024) Oxidative stress in metabolic dysfunction-associated steatotic liver disease (MASLD): How does the animal model resemble human disease? FASEB J 38(3):e23466 doi:10.1096/fj.202302447R

Jarukamjorn K, Jearapong N, Pimson C, Chatuphonprasert W (2016) A High-Fat, High-Fructose Diet Induces Antioxidant Imbalance and Increases the Risk and Progression of Nonalcoholic Fatty Liver Disease in Mice. Scientifica (Cairo) 2016:5029414 doi:10.1155/2016/5029414

Jiang J, Li H, Tang M, et al. (2024) Upregulation of Hepatic Glutathione S-Transferase Alpha 1 Ameliorates Metabolic Dysfunction-Associated Steatosis by Degrading Fatty Acid Binding Protein 1. International journal of molecular sciences 25(10) doi:10.3390/ijms25105086

Jin M, Wei Y, Yu H, et al. (2021) Erythritol Improves Nonalcoholic Fatty Liver Disease by Activating Nrf2 Antioxidant Capacity. J Agric Food Chem 69(44):13080-13092 doi:10.1021/acs.jafc.1c05213

Jorgačević B, Mladenović D, Ninković M, et al. (2014) Dynamics of oxidative/nitrosative stress in mice with methionine-choline-deficient diet-induced nonalcoholic fatty liver disease. Hum Exp Toxicol 33(7):701-9 doi:10.1177/0960327113506723

Jung JH, Kim HS (2013) The inhibitory effect of black soybean on hepatic cholesterol accumulation in high cholesterol and high fat diet-induced non-alcoholic fatty liver disease. Food Chem Toxicol 60:404-12 doi:10.1016/j.fct.2013.07.048

Kanoni S, Kumar S, Amerikanou C, et al. (2021) Nutrigenetic Interactions Might Modulate the Antioxidant and Anti-Inflammatory Status in Mastiha-Supplemented Patients With NAFLD. Front Immunol 12:683028 doi:10.3389/fimmu.2021.683028

Ke Z, Tan S, Li H, et al. (2022) Tangeretin improves hepatic steatosis and oxidative stress through the Nrf2 pathway in high fat diet-induced nonalcoholic fatty liver disease mice. Food Funct 13(5):2782-2790 doi:10.1039/d1fo02989d

Kim HJ, Lee Y, Fang S, Kim W, Kim HJ, Kim JW (2020) GPx7 ameliorates non-alcoholic steatohepatitis by regulating oxidative stress. BMB Rep 53(6):317-322 doi:10.5483/BMBRep.2020.53.6.280

Kim JW, Lee YS, Seol DJ, et al. (2018) Anti-obesity and fatty liver-preventing activities of Lonicera caerulea in high-fat diet-fed mice. Int J Mol Med 42(6):3047-3064 doi:10.3892/ijmm.2018.3879

Kohjima M, Enjoji M, Higuchi N, et al. (2007) Re-evaluation of fatty acid metabolism-related gene expression in nonalcoholic fatty liver disease. Int J Mol Med 20(3):351-8

Korish AA, Arafah MM (2013) Camel milk ameliorates steatohepatitis, insulin resistance and lipid peroxidation in experimental non-alcoholic fatty liver disease. BMC Complement Altern Med 13:264 doi:10.1186/1472-6882-13-264

Koruk M, Taysi S, Savas MC, Yilmaz O, Akcay F, Karakok M (2004) Oxidative stress and enzymatic antioxidant status in patients with nonalcoholic steatohepatitis. Ann Clin Lab Sci 34(1):57-62

Krishnan A, Abdullah TS, Mounajjed T, et al. (2017) A longitudinal study of whole body, tissue, and cellular physiology in a mouse model of fibrosing NASH with high fidelity to the human condition. Am J Physiol Gastrointest Liver Physiol 312(6):G666-g680 doi:10.1152/ajpgi.00213.2016

Kumar A, Sharma A, Duseja A, et al. (2013) Patients with Nonalcoholic Fatty Liver Disease (NAFLD) have Higher Oxidative Stress in Comparison to Chronic Viral Hepatitis. J Clin Exp Hepatol 3(1):12-8 doi:10.1016/j.jceh.2012.10.009

Lee CY, Lee CL (2021) Comparison of the Improvement Effect of Deep Ocean Water with Different Mineral Composition on the High Fat Diet-Induced Blood Lipid and Nonalcoholic Fatty Liver Disease in a Mouse Model. Nutrients 13(5) doi:10.3390/nu13051732

Lee DH, Han DH, Nam KT, et al. (2016) Ezetimibe, an NPC1L1 inhibitor, is a potent Nrf2 activator that protects mice from diet-induced nonalcoholic steatohepatitis. Free radical biology & medicine 99:520-532 doi:10.1016/j.freeradbiomed.2016.09.009

Li H, Toth E, Cherrington NJ (2018) Alcohol Metabolism in the Progression of Human Nonalcoholic Steatohepatitis. Toxicol Sci 164(2):428-438 doi:10.1093/toxsci/kfy106

Li J, Wang T, Liu P, et al. (2021) Hesperetin ameliorates hepatic oxidative stress and inflammation via the PI3K/AKT-Nrf2-ARE pathway in oleic acid-induced HepG2 cells and a rat model of high-fat diet-induced NAFLD. Food Funct 12(9):3898-3918 doi:10.1039/d0fo02736g

Li L, Hai J, Li Z, et al. (2014) Resveratrol modulates autophagy and NF-κB activity in a murine model for treating non-alcoholic fatty liver disease. Food Chem Toxicol 63:166-73 doi:10.1016/j.fct.2013.08.036

Li N, Sun YR, He LB, et al. (2020) Amelioration by Idesia polycarpa Maxim. var. vestita Diels. of Oleic Acid-Induced Nonalcoholic Fatty Liver in HepG2 Cells through Antioxidant and Modulation of Lipid Metabolism. Oxidative medicine and cellular longevity 2020:1208726 doi:10.1155/2020/1208726

Li W, Lu Y (2018) Hepatoprotective Effects of Sophoricoside against Fructose-Induced Liver Injury via Regulating Lipid Metabolism, Oxidation, and Inflammation in Mice. J Food Sci 83(2):552-558 doi:10.1111/1750-3841.14047

Lickteig AJ, Fisher CD, Augustine LM, Cherrington NJ (2007) Genes of the antioxidant response undergo upregulation in a rodent model of nonalcoholic steatohepatitis. J Biochem Mol Toxicol 21(4):216-20 doi:10.1002/jbt.20177

Liu YT, Chen HW, Lii CK, et al. (2020) A Diterpenoid, 14-Deoxy-11, 12-Didehydroandrographolide, in Andrographis paniculata Reduces Steatohepatitis and Liver Injury in Mice Fed a High-Fat and High-Cholesterol Diet. Nutrients 12(2) doi:10.3390/nu12020523

Longhitano L, Distefano A, Musso N, et al. (2024) (+)-Lipoic acid reduces mitochondrial unfolded protein response and attenuates oxidative stress and aging in an in vitro model of non-alcoholic fatty liver disease. J Transl Med 22(1):82 doi:10.1186/s12967-024-04880-x

Lu JH, Hsia K, Lin CH, Chen CC, Yang HY, Lin MH (2019) Dietary Supplementation with Hazelnut Oil Reduces Serum Hyperlipidemia and Ameliorates the Progression of Nonalcoholic Fatty Liver Disease in Hamsters Fed a High-Cholesterol Diet. Nutrients 11(9) doi:10.3390/nu11092224

Ma C, Liu Y, He S, et al. (2020) Association Between Leukocyte Mitochondrial DNA Copy Number and Non-alcoholic Fatty Liver Disease in a Chinese Population Is Mediated by 8-Oxo-2'-Deoxyguanosine. Front Med (Lausanne) 7:536 doi:10.3389/fmed.2020.00536

Malaguarnera L, Madeddu R, Palio E, Arena N, Malaguarnera M (2005) Heme oxygenase-1 levels and oxidative stress-related parameters in non-alcoholic fatty liver disease patients. Journal of hepatology 42(4):585-91 doi:10.1016/j.jhep.2004.11.040

Marinho PC, Vieira AB, Pereira PG, et al. (2018) Capybara Oil Improves Hepatic Mitochondrial Dysfunction, Steatosis, and Inflammation in a Murine Model of Nonalcoholic Fatty Liver Disease. Evidence-based complementary and alternative medicine : eCAM 2018:4956079 doi:10.1155/2018/4956079

Matouskova P, Bartikova H, Bousova I, Levorova L, Szotakova B, Skalova L (2015) Drug-metabolizing and antioxidant enzymes in monosodium L-glutamate obese mice. Drug Metab Dispos 43(2):258-65 doi:10.1124/dmd.114.061176

Mendes IKS, Matsuura C, Aguila MB, et al. (2018) Weight loss enhances hepatic antioxidant status in a NAFLD model induced by high-fat diet. Appl Physiol Nutr Metab 43(1):23-29 doi:10.1139/apnm-2017-0317

Monserrat-Mesquida M, Quetglas-Llabrés M, Abbate M, et al. (2020) Oxidative Stress and Pro-Inflammatory Status in Patients with Non-Alcoholic Fatty Liver Disease. Antioxidants (Basel) 9(8) doi:10.3390/antiox9080759

Moya D, Baker SS, Liu W, et al. (2015) Novel pathway for iron deficiency in pediatric non-alcoholic steatohepatitis. Clin Nutr 34(3):549-56 doi:10.1016/j.clnu.2014.06.011

Murakami S, Ono A, Kawasaki A, Takenaga T, Ito T (2018) Taurine attenuates the development of hepatic steatosis through the inhibition of oxidative stress in a model of nonalcoholic fatty liver disease in vivo and in vitro. Amino acids 50(9):1279-1288 doi:10.1007/s00726-018-2605-8

Nagaya T, Tanaka N, Kimura T, et al. (2015) Mechanism of the development of nonalcoholic steatohepatitis after pancreaticoduodenectomy. BBA Clin 3:168-74 doi:10.1016/j.bbacli.2015.02.001

Nam H, Lim JH, Kim TW, et al. (2023) Extracellular Superoxide Dismutase Attenuates Hepatic Oxidative Stress in Nonalcoholic Fatty Liver Disease through the Adenosine Monophosphate-Activated Protein Kinase Activation. Antioxidants (Basel) 12(12) doi:10.3390/antiox12122040

Nayan SI, Chowdhury FI, Akter N, et al. (2021) Leaf powder supplementation of Senna alexandrina ameliorates oxidative stress, inflammation, and hepatic steatosis in high-fat diet-fed obese rats. PLoS One 16(4):e0250261 doi:10.1371/journal.pone.0250261

Nobili V, Pastore A, Gaeta LM, et al. (2005) Glutathione metabolism and antioxidant enzymes in patients affected by nonalcoholic steatohepatitis. Clin Chim Acta 355(1-2):105-11 doi:10.1016/j.cccn.2004.12.022

Noeman SA, Hamooda HE, Baalash AA (2011) Biochemical study of oxidative stress markers in the liver, kidney and heart of high fat diet induced obesity in rats. Diabetology & metabolic syndrome 3(1):17 doi:10.1186/1758-5996-3-17

Nosrati N, Aghazadeh S, Yazdanparast R (2010) Effects of Teucrium polium on Insulin Resistance in Nonalcoholic Steatohepatitis. J Acupunct Meridian Stud 3(2):104-10 doi:10.1016/s2005-2901(10)60019-2

Nunes-Souza V, César-Gomes CJ, Da Fonseca LJ, Guedes Gda S, Smaniotto S, Rabelo LA (2016) Aging Increases Susceptibility to High Fat Diet-Induced Metabolic Syndrome in C57BL/6 Mice: Improvement in Glycemic and Lipid Profile after Antioxidant Therapy. Oxidative medicine and cellular longevity 2016:1987960 doi:10.1155/2016/1987960

Omogbiya AI, Ben-Azu B, Eduviere AT, et al. (2021) Monosodium glutamate induces memory and hepatic dysfunctions in mice: ameliorative role of Jobelyn((R)) through the augmentation of cellular antioxidant defense machineries. Toxicol Res 37(3):323-335 doi:10.1007/s43188-020-00068-9

Ortenzi VH, Oliveira AC, Vasconcelos RP, et al. (2024) High-fat diet elicits sex-based differences in liver inflammatory cytokines and redox homeostasis. Appl Physiol Nutr Metab 49(8):1083-1092 doi:10.1139/apnm-2023-0457

Pacana T, Cazanave S, Verdianelli A, et al. (2015) Dysregulated Hepatic Methionine Metabolism Drives Homocysteine Elevation in Diet-Induced Nonalcoholic Fatty Liver Disease. PLoS One 10(8):e0136822 doi:10.1371/journal.pone.0136822

Park HS, Jang JE, Ko MS, et al. (2016) Statins Increase Mitochondrial and Peroxisomal Fatty Acid Oxidation in the Liver and Prevent Non-Alcoholic Steatohepatitis in Mice. Diabetes Metab J 40(5):376-385 doi:10.4093/dmj.2016.40.5.376

Pereira E, Silvares RR, Flores EEI, et al. (2017) Hepatic microvascular dysfunction and increased advanced glycation end products are components of non-alcoholic fatty liver disease. PLoS One 12(6):e0179654 doi:10.1371/journal.pone.0179654

Perlemuter G, Davit-Spraul A, Cosson C, et al. (2005) Increase in liver antioxidant enzyme activities in non-alcoholic fatty liver disease. Liver Int 25(5):946-53 doi:10.1111/j.1478-3231.2005.01126.x

Rodriguez-Ramiro I, Pastor-Fernandez A, Lopez-Aceituno JL, et al. (2024) Pharmacological and genetic increases in liver NADPH levels ameliorate NASH progression in female mice. Free radical biology & medicine 210:448-461 doi:10.1016/j.freeradbiomed.2023.11.019

Rosas-Campos R, Sandoval-Rodriguez AS, Rodriguez-Sanabria JS, et al. (2024) A Novel Foodstuff Mixture Improves the Gut-Liver Axis in MASLD Mice and the Gut Microbiota in Overweight/Obese Patients. Antioxidants (Basel) 13(6) doi:10.3390/antiox13060664

Sabir U, Irfan HM, Alamgeer, Ullah A, Althobaiti YS, Asim MH (2022) Reduction of Hepatic Steatosis, Oxidative Stress, Inflammation, Ballooning and Insulin Resistance After Therapy with Safranal in NAFLD Animal Model: A New Approach. J Inflamm Res 15:1293-1316 doi:10.2147/jir.S354878

Santamaria E, Avila MA, Latasa MU, et al. (2003) Functional proteomics of nonalcoholic steatohepatitis: mitochondrial proteins as targets of S-adenosylmethionine. Proceedings of the National Academy of Sciences of the United States of America 100(6):3065-70 doi:10.1073/pnas.0536625100

Santos-López JA, Garcimartín A, Merino P, et al. (2016) Effects of Silicon vs. Hydroxytyrosol-Enriched Restructured Pork on Liver Oxidation Status of Aged Rats Fed High-Saturated/High-Cholesterol Diets. PLoS One 11(1):e0147469 doi:10.1371/journal.pone.0147469

Santos JDB, Mendonça AAS, Sousa RC, et al. (2018) Food-drug interaction: Anabolic steroids aggravate hepatic lipotoxicity and nonalcoholic fatty liver disease induced by trans fatty acids. Food Chem Toxicol 116(Pt B):360-368 doi:10.1016/j.fct.2018.04.056

Sharma A, Anand SK, Singh N, Dwivedi UN, Kakkar P (2020) Berbamine induced AMPK activation regulates mTOR/SREBP-1c axis and Nrf2/ARE pathway to allay lipid accumulation and oxidative stress in steatotic HepG2 cells. Eur J Pharmacol 882:173244 doi:10.1016/j.ejphar.2020.173244

Shatoor AS, Al Humayed S, Almohiy HM (2021) Crataegus aronia prevents high-fat diet-induced hepatic steatosis in rats by activating AMPK-induced suppression of SREBP1 and activation of PPARα. J Food Biochem 45(11):e13945 doi:10.1111/jfbc.13945

Shearn CT, Saba LM, Roede JR, Orlicky DJ, Shearn AH, Petersen DR (2017) Differential carbonylation of proteins in end-stage human fatty and nonfatty NASH. Free radical biology & medicine 113:280-290 doi:10.1016/j.freeradbiomed.2017.10.004

Shin SK, Cho HW, Song SE, Song DK (2018) Catalase and nonalcoholic fatty liver disease. Pflugers Arch 470(12):1721-1737 doi:10.1007/s00424-018-2195-z

Silja K, Selvaganabathy N, Kalaiselvi T, Thirunavukkarasu C (2023) Inhibition of glutathione generation in hepatic steatotic rats augments oxidative stress. Toxicol Mech Methods 33(7):596-606 doi:10.1080/15376516.2023.2202784

Song L, Qu D, Zhang Q, et al. (2017) Phytosterol esters attenuate hepatic steatosis in rats with non-alcoholic fatty liver disease rats fed a high-fat diet. Sci Rep 7:41604 doi:10.1038/srep41604

Sreekumar R, Rosado B, Rasmussen D, Charlton M (2003) Hepatic gene expression in histologically progressive nonalcoholic steatohepatitis. Hepatology 38(1):244-51 doi:10.1053/jhep.2003.50290

Stefano JT, Pereira IV, Torres MM, et al. (2015) Sorafenib prevents liver fibrosis in a non-alcoholic steatohepatitis (NASH) rodent model. Braz J Med Biol Res 48(5):408-14 doi:10.1590/1414-431x20143962

Su H, Li Y, Hu D, et al. (2018) Procyanidin B2 ameliorates free fatty acids-induced hepatic steatosis through regulating TFEB-mediated lysosomal pathway and redox state. Free radical biology & medicine 126:269-286 doi:10.1016/j.freeradbiomed.2018.08.024

Świderska M, Maciejczyk M, Zalewska A, Pogorzelska J, Flisiak R, Chabowski A (2019) Oxidative stress biomarkers in the serum and plasma of patients with non-alcoholic fatty liver disease (NAFLD). Can plasma AGE be a marker of NAFLD? Oxidative stress biomarkers in NAFLD patients. Free Radic Res 53(8):841-850 doi:10.1080/10715762.2019.1635691

Tanaka K, Masaki Y, Tanaka M, et al. (2014) Exenatide improves hepatic steatosis by enhancing lipid use in adipose tissue in nondiabetic rats. World J Gastroenterol 20(10):2653-63 doi:10.3748/wjg.v20.i10.2653

Thomàs-Moyà E, Gómez-Pérez Y, Fiol M, Gianotti M, Lladó I, Proenza AM (2008) Gender related differences in paraoxonase 1 response to high-fat diet-induced oxidative stress. Obesity 16(10):2232-8 doi:10.1038/oby.2008.340

Tsai SY, Chung PC, Owaga EE, et al. (2016) Alpha-mangostin from mangosteen (Garcinia mangostana Linn.) pericarp extract reduces high fat-diet induced hepatic steatosis in rats by regulating mitochondria function and apoptosis. Nutr Metab (Lond) 13:88 doi:10.1186/s12986-016-0148-0

Valdecantos MP, Pérez-Matute P, González-Muniesa P, Prieto-Hontoria PL, Moreno-Aliaga MJ, Martínez JA (2012) Lipoic acid improves mitochondrial function in nonalcoholic steatosis through the stimulation of sirtuin 1 and sirtuin 3. Obesity 20(10):1974-83 doi:10.1038/oby.2012.32

Valenzuela R, Echeverria F, Ortiz M, et al. (2017) Hydroxytyrosol prevents reduction in liver activity of Δ-5 and Δ-6 desaturases, oxidative stress, and depletion in long chain polyunsaturated fatty acid content in different tissues of high-fat diet fed mice. Lipids Health Dis 16(1):64 doi:10.1186/s12944-017-0450-5

Valenzuela R, Rincón-Cervera M, Echeverría F, et al. (2018) Iron-induced pro-oxidant and pro-lipogenic responses in relation to impaired synthesis and accretion of long-chain polyunsaturated fatty acids in rat hepatic and extrahepatic tissues. Nutrition 45:49-58 doi:10.1016/j.nut.2017.07.007

Vecchione G, Grasselli E, Voci A, et al. (2016) Silybin counteracts lipid excess and oxidative stress in cultured steatotic hepatic cells. World J Gastroenterol 22(26):6016-26 doi:10.3748/wjg.v22.i26.6016

Veeramani C, Alsaif MA, Al-Numair KS (2017) Lavatera critica, a green leafy vegetable, controls high fat diet induced hepatic lipid accumulation and oxidative stress through the regulation of lipogenesis and lipolysis genes. Biomed Pharmacother 96:1349-1357 doi:10.1016/j.biopha.2017.11.072

Videla LA, Rodrigo R, Orellana M, et al. (2004) Oxidative stress-related parameters in the liver of non-alcoholic fatty liver disease patients. Clin Sci (Lond) 106(3):261-8 doi:10.1042/cs20030285

Vornoli A, Pozzo L, Della Croce CM, Gervasi PG, Longo V (2014) Drug metabolism enzymes in a steatotic model of rat treated with a high fat diet and a low dose of streptozotocin. Food Chem Toxicol 70:54-60 doi:10.1016/j.fct.2014.04.042

Wang DJ, Cai YQ, Pan SZ, et al. (2018) Effect of Total Flavone of Haw Leaves on Nuclear Factor Erythroid-2 Related Factor and Other Related Factors in Nonalcoholic Steatohepatitis Rats. Chin J Integr Med 24(4):265-271 doi:10.1007/s11655-016-2450-0

Wang Y, Chen J, Kong W, et al. (2017) Regulation of SIRT3/FOXO1 Signaling Pathway in Rats with Non-alcoholic Steatohepatitis by Salvianolic Acid B. Arch Med Res 48(6):506-512 doi:10.1016/j.arcmed.2017.11.016

Wat E, Ng CF, Wong EC, et al. (2016) The hepatoprotective effect of the combination use of Fructus Schisandrae with statin--A preclinical evaluation. Journal of ethnopharmacology 178:104-14 doi:10.1016/j.jep.2015.12.004

Wu D, Zheng N, Qi K, et al. (2015) Exogenous hydrogen sulfide mitigates the fatty liver in obese mice through improving lipid metabolism and antioxidant potential. Med Gas Res 5(1):1 doi:10.1186/s13618-014-0022-y

Wu PJ, Chen JB, Lee WC, et al. (2018) Oxidative Stress and Nonalcoholic Fatty Liver Disease in Hemodialysis Patients. Biomed Res Int 2018:3961748 doi:10.1155/2018/3961748

Xia SF, Le GW, Wang P, Qiu YY, Jiang YY, Tang X (2016) Regressive Effect of Myricetin on Hepatic Steatosis in Mice Fed a High-Fat Diet. Nutrients 8(12) doi:10.3390/nu8120799

Xia SF, Shao J, Zhao SY, et al. (2018) Niga-ichigoside F1 ameliorates high-fat diet-induced hepatic steatosis in male mice by Nrf2 activation. Food Funct 9(2):906-916 doi:10.1039/c7fo01051f

Xie K, He X, Chen K, Sakao K, Hou DX (2020) Ameliorative effects and molecular mechanisms of vine tea on western diet-induced NAFLD. Food Funct 11(7):5976-5991 doi:10.1039/d0fo00795a

Xu J, Wang X, Cao K, Dong Z, Feng Z, Liu J (2017) Combination of β-glucan and Morus alba L. Leaf Extract Promotes Metabolic Benefits in Mice Fed a High-Fat Diet. Nutrients 9(10) doi:10.3390/nu9101110

Xu X, Sun S, Liang L, et al. (2021) Role of the Aryl Hydrocarbon Receptor and Gut Microbiota-Derived Metabolites Indole-3-Acetic Acid in Sulforaphane Alleviates Hepatic Steatosis in Mice. Front Nutr 8:756565 doi:10.3389/fnut.2021.756565

Yang ZR, Wang HF, Zuo TC, Guan LL, Dai N (2016) Salidroside alleviates oxidative stress in the liver with non- alcoholic steatohepatitis in rats. BMC Pharmacol Toxicol 17:16 doi:10.1186/s40360-016-0059-8

Ye J, Tian X, Wang Q, et al. (2022) Monkfish Peptides Mitigate High Fat Diet-Induced Hepatic Steatosis in Mice. Mar Drugs 20(5) doi:10.3390/md20050312

Ye M, Tang Y, He J, et al. (2021) Alleviation of non-alcoholic fatty liver disease by Huazhi Fugan Granules is associated with suppression of TLR4/NF-κB signaling pathway. Clin Investig Arterioscler 33(5):257-266 doi:10.1016/j.arteri.2020.12.007

Ye Q, Jiang Y, Wu D, et al. (2023) Atractylodin alleviates nonalcoholic fatty liver disease by regulating Nrf2-mediated ferroptosis. Heliyon 9(7):e18321 doi:10.1016/j.heliyon.2023.e18321

Yesilova Z, Yaman H, Oktenli C, et al. (2005) Systemic markers of lipid peroxidation and antioxidants in patients with nonalcoholic Fatty liver disease. Am J Gastroenterol 100(4):850-5 doi:10.1111/j.1572-0241.2005.41500.x

Yoshioka S, Hamada A, Jobu K, et al. (2010) Effects of Eriobotrya japonica seed extract on oxidative stress in rats with non-alcoholic steatohepatitis. J Pharm Pharmacol 62(2):241-6 doi:10.1211/jpp.62.02.0012

Younossi ZM, Baranova A, Ziegler K, et al. (2005) A genomic and proteomic study of the spectrum of nonalcoholic fatty liver disease. Hepatology 42(3):665-74 doi:10.1002/hep.20838

Yu Q, Lee YY, Xia ZY, Liong EC, Xiao J, Tipoe GL (2021) S-allylmercaptocysteine improves nonalcoholic steatohepatitis by enhancing AHR/NRF2-mediated drug metabolising enzymes and reducing NF-κB/IκBα and NLRP3/6-mediated inflammation. European journal of nutrition 60(2):961-973 doi:10.1007/s00394-020-02305-1

Zakaria Z, Othman ZA, Suleiman JB, et al. (2021) Hepatoprotective Effect of Bee Bread in Metabolic Dysfunction-Associated Fatty Liver Disease (MAFLD) Rats: Impact on Oxidative Stress and Inflammation. Antioxidants (Basel) 10(12) doi:10.3390/antiox10122031

Zhang T, Wang MY, Wang GD, et al. (2024) Metformin improves nonalcoholic fatty liver disease in db/db mice by inhibiting ferroptosis. Eur J Pharmacol 966:176341 doi:10.1016/j.ejphar.2024.176341

Zhang X, Ji R, Sun H, et al. (2018) Scutellarin ameliorates nonalcoholic fatty liver disease through the PPARγ/PGC-1α-Nrf2 pathway. Free Radic Res 52(2):198-211 doi:10.1080/10715762.2017.1422602

Zhao MG, Sheng XP, Huang YP, et al. (2018a) Triterpenic acids-enriched fraction from Cyclocarya paliurus attenuates non-alcoholic fatty liver disease via improving oxidative stress and mitochondrial dysfunction. Biomed Pharmacother 104:229-239 doi:10.1016/j.biopha.2018.03.170

Zhao XJ, Yu HW, Yang YZ, et al. (2018b) Polydatin prevents fructose-induced liver inflammation and lipid deposition through increasing miR-200a to regulate Keap1/Nrf2 pathway. Redox biology 18:124-137 doi:10.1016/j.redox.2018.07.002

Zheng W, Song Z, Li S, Hu M, Shaukat H, Qin H (2021) Protective Effects of Sesamol against Liver Oxidative Stress and Inflammation in High-Fat Diet-Induced Hepatic Steatosis. Nutrients 13(12) doi:10.3390/nu13124484

Zhu CG, Liu YX, Wang H, et al. (2017) Active form of vitamin D ameliorates non-alcoholic fatty liver disease by alleviating oxidative stress in a high-fat diet rat model. Endocr J 64(7):663-673 doi:10.1507/endocrj.EJ16-0542

Zhu Z, Hu R, Li J, et al. (2021) Alpinetin exerts anti-inflammatory, anti-oxidative and anti-angiogenic effects through activating the Nrf2 pathway and inhibiting NLRP3 pathway in carbon tetrachloride-induced liver fibrosis. Int Immunopharmacol 96:107660 doi:10.1016/j.intimp.2021.107660

Zilu S, Qian H, Haibin W, et al. (2019) Effects of XIAP on high fat diet-induced hepatic steatosis: a mechanism involving NLRP3 inflammasome and oxidative stress. Aging (Albany NY) 11(24):12177-12201 doi:10.18632/aging.102559
